# Implementation of an automated workflow for image-based seafloor classification with examples from manganese-nodule covered seabed areas in the Central Pacific Ocean

**DOI:** 10.1038/s41598-022-19070-2

**Published:** 2022-09-12

**Authors:** Benson Mbani, Timm Schoening, Iason-Zois Gazis, Reinhard Koch, Jens Greinert

**Affiliations:** 1 DeepSea Monitoring Group, GEOMAR Helmholtz Center for Ocean Research Kiel, Wischhofstraße 1-3, 24148 Kiel, Germany; 2 Department of Computer Science, Kiel University, Olshausenstr. 40, 24098 Kiel, Germany; 3 Institute of Geosciences, Kiel University, Ludewig-Meyn-Str. 10, 24118 Kiel, Germany

**Keywords:** Ocean sciences, Computer science

## Abstract

Mapping and monitoring of seafloor habitats are key tasks for fully understanding ocean ecosystems and resilience, which contributes towards sustainable use of ocean resources. Habitat mapping relies on seafloor classification typically based on acoustic methods, and ground truthing through direct sampling and optical imaging. With the increasing capabilities to record high-resolution underwater images, manual approaches for analyzing these images to create seafloor classifications are no longer feasible. Automated workflows have been proposed as a solution, in which algorithms assign pre-defined seafloor categories to each image. However, in order to provide consistent and repeatable analysis, these automated workflows need to address e.g., underwater illumination artefacts, variances in resolution and class-imbalances, which could bias the classification. Here, we present a generic implementation of an Automated and Integrated Seafloor Classification Workflow (AI-SCW). The workflow aims to classify the seafloor into habitat categories based on automated analysis of optical underwater images with only minimal amount of human annotations. AI-SCW incorporates laser point detection for scale determination and color normalization. It further includes semi-automatic generation of the training data set for fitting the seafloor classifier. As a case study, we applied the workflow to an example seafloor image dataset from the Belgian and German contract areas for Manganese-nodule exploration in the Pacific Ocean. Based on this, we provide seafloor classifications along the camera deployment tracks, and discuss results in the context of seafloor multibeam bathymetry. Our results show that the seafloor in the Belgian area predominantly comprises densely distributed nodules, which are intermingled with qualitatively larger-sized nodules at local elevations and within depressions. On the other hand, the German area primarily comprises nodules that only partly cover the seabed, and these occur alongside turned-over sediment (artificial seafloor) that were caused by the settling plume following a dredging experiment conducted in the area.

## Introduction

Understanding the geological and ecological characteristics of the seafloor is key for monitoring and managing marine ecosystems. Together with bathymetric information, underwater optical images are typically used to characterize the seafloor by partitioning it into classes, and assigning the relevant semantic seafloor- or habitat-label to each class^1^. The cameras recording these images are usually attached to platforms such as towed camera frames as in the Ocean Floor Observation System (OFOS), Automated Underwater Vehicles (AUVs) and Remotely Operated Vehicles (ROVs). Such cameras generate a high-volume of seafloor images in number and storage space, whose analysis requires automated workflows. This is because manually inspecting each photo is infeasible and non-scalable^2^ and thus, automated seafloor classification workflows are preferred because of the benefit of repeatability, which also reduces subjectivity and bias^3^. Despite this benefit, the implementation of automated workflows is still challenging. This is on one hand because of changing conditions during image acquisition even within one dive (changing light conditions from different altitude; variable backscatter of particles in the water over time) and between dives (potentially different light and camera configurations), but also because domain expertise is needed to annotate a large number of training examples for fitting an image classification model. This necessitates the implementation of seafloor classification workflows, which require only minimal human annotations when dealing with a voluminous dataset comprising images recorded with same camera and illuminating system. When the trained image classification models are used to make automated predictions, they assist in rather quick and objective generation of baseline habitat information, which is crucial for managing marine ecosystems and potentially man-made changes in general^3^, but also in adjusting sampling strategies on the fly.

During image acquisition, laser pointers mounted around the camera build a fixed calibrated geometry that is used for scale determination of the image. Two or more laser points are photographed to define e.g., a triangular geometry of red dots around the center of each acquired photo. The pixel-distance of the photographed geometry in each image varies in proportion to the camera altitude above the seafloor. The ratio between the photographed and calibrated geometries can be used to infer both the image scale and approximate altitude of each photo during acquisition. However, the varying size of the photographed geometry, and changes of the seafloor color and roughness makes it challenging to automatically detect the laser points by existing techniques such as template matching e.g. as described in^4^. Another challenge is that each laser point may only be represented in a 20 pixel area^5^, which is small and cannot be detected by standard object detection algorithms such as single shot multi-box detector^6^ or YOLO^7^. Despite these challenges, the image scale is essential for downstream tasks, such as illumination normalization, and conversion of measurements from pixel units to metric units^5^. This necessitates the incorporation of a laser point detection into the seafloor classification workflow, which is invariant to the mentioned challenges.

Usually, a significant proportion of photos recorded during video transect deployments are redundant for wider area seafloor or habitat mapping purposes, because the camera records a photo e.g., every 10th or even every second, while being towed at slow speed of 0.5 knots (~ 0.9 m/s; every 10 m or 1 m an image). Therefore, the same habitat/seafloor type is likely to be photographed in multiple frames. Particularly for the deep sea, most photos represent the dominant seafloor class since the deep sea is changing slowly in space (hundreds of meters). However, this might be different in shallow water where the geological substrate such as rocks, sand, or outcropping basement may change quickly on meter scale. In the deep sea, the more homogenous seafloor causes a class imbalance, since the frequency of photos representing the dominant seafloor classes is disproportionately higher than the rare classes^8^. A classification model trained on this imbalanced dataset is likely to be biased, by falsely associating photos with the dominant class, causing lower classification performance. In addition, the high number of images recorded during an expedition causes computational bottlenecks when used to train a seafloor classifier, because storing the images requires large computer memory and computational resources^9^. These challenges need to be addressed by an automated classification workflow. This can be done by identifying an efficient sampling strategy, which generates class-balanced training set that fits in computer memory during the training of a classification model.

The training of a seafloor classifier can either be supervised or unsupervised. It is considered supervised when manually labeled images are used, and unsupervised otherwise. Even though supervised classifiers perform better when trained with sufficiently labeled examples^10^, manually annotating the images is a subjective and time-consuming process that has limitations for very large data sets^11^. This calls for semi-automated annotation workflows, in which a human analyst provides only limited labels, while the rest are automatically generated. Alternatively, a fully unsupervised classification model could be trained to classify the seafloor by clustering unlabeled photos. A human analyst is then required to only assign semantic labels to the resulting clusters. Therefore, an automated classification workflow should incorporate both supervised and unsupervised classification approaches, so that users can choose whichever is more convenient.

Past studies on automated laser point detection mostly involve manually annotating a set of laser points to be used for training either a classifier or regression model, which is then used to automatically detect laser points on test images^5,12^. While these approaches work well, they also require a lot of manual annotation which renders them sometimes infeasible. With respect to seafloor classification, there have also been previous implementations using different approaches. Most workflows typically employ technologies such as side scan sonar and multi-beam echosounders to record hydro acoustic or multispectral imagery at kilometer scale, after which remote sensing based image interpretation techniques are used to discriminate among the different benthic habitat categories using site specific fingerprints^13–15^. Such acoustic-based methods have also been previously developed by established institutions e.g. MBARI’s integrated mapping system^16^, IFREMER’s software for multibeam echosounder and side scan sonar mapping (CARAIBES)^17^ and JAMSTEC’s deep seafloor monitoring system^18^. The limiting characteristic of these hydro-acoustic based workflows is that their footprint size makes them unsuitable for detecting small objects on the seafloor. This limitation is addressed when processing optical images with millimeter resolution. Previous studies that derived seafloor classes from images involved the use of hand engineered features such as color and texture to encode visual content from image patches, upon which supervised and unsupervised classifiers are trained^19–22^. Further, there have been studies that employed photogrammetric techniques to monitor the benthic habitat coverage using orthophoto mosaics and dense point clouds^23^. These approaches assume that discernible features e.g., texture and color extracted from underwater photos are enough information to distinguish the various seafloor categories. To avoid this assumption, other studies employ deep learning to automatically extract abstract features using a sequence of convolution and pooling operations^24,25^. A linear classifier fit on top of these features is then used to assign each image to a habitat class. The drawback of these deep learning approaches is, that they require substantial manually annotated training examples, without which they easily lead to overfitting^26^.

With the above given setting in mind, we develop a generic end-to-end workflow that aims to assist marine researchers to automate image-based seafloor classification. The seafloor classification workflow (AI-SCW) works on optical RGB images recorded by any platform such as ROV, AUV and towed camera systems as the OFOS. The workflow starts with automatically detecting laser points and using these to infer the photo scale. The scale is used as the basis for choosing e.g., a reference image for color normalization. This normalization is done by matching the histogram of all other images to the respective reference image. The images are further rescaled to a defined resolution, and central parts of the image are cropped out to have the same spatial footprint in square meters. The cropping also cuts off the darker image rims that are typical for deep sea images as a result of too little light. A labeled training set is then generated semi-automatically, and used to fit a convolutional neural network classification model using the Inception V3 architecture^27^. In addition to this approach, an empirical comparison of four sampling strategies is done to identify the optimal strategy to generate training data for fitting an unsupervised k-means classification model.

Although we use an example of a Mn-nodule area in the deep sea because of the current economic and ecological interest for such areas, AI-SCW can be used to classify any other seafloor. Thus, it is useful to researchers in the marine community who routinely use optical imagery to monitor the seafloor geology, ecosystem or habitats, and who aim for an automated and less subjective image analysis.

## Results

### Image pre-processing; scale determination and color correction

This section presents the results of automated laser point detection and subsequent determination of scale for all the underwater images. Also presented are results for the correction of artifacts caused by illumination drop-off towards the periphery of the images, and also color normalization to correct for uneven scene brightness among the images.

#### Laser point detection and scale determination

**Figure 1:**
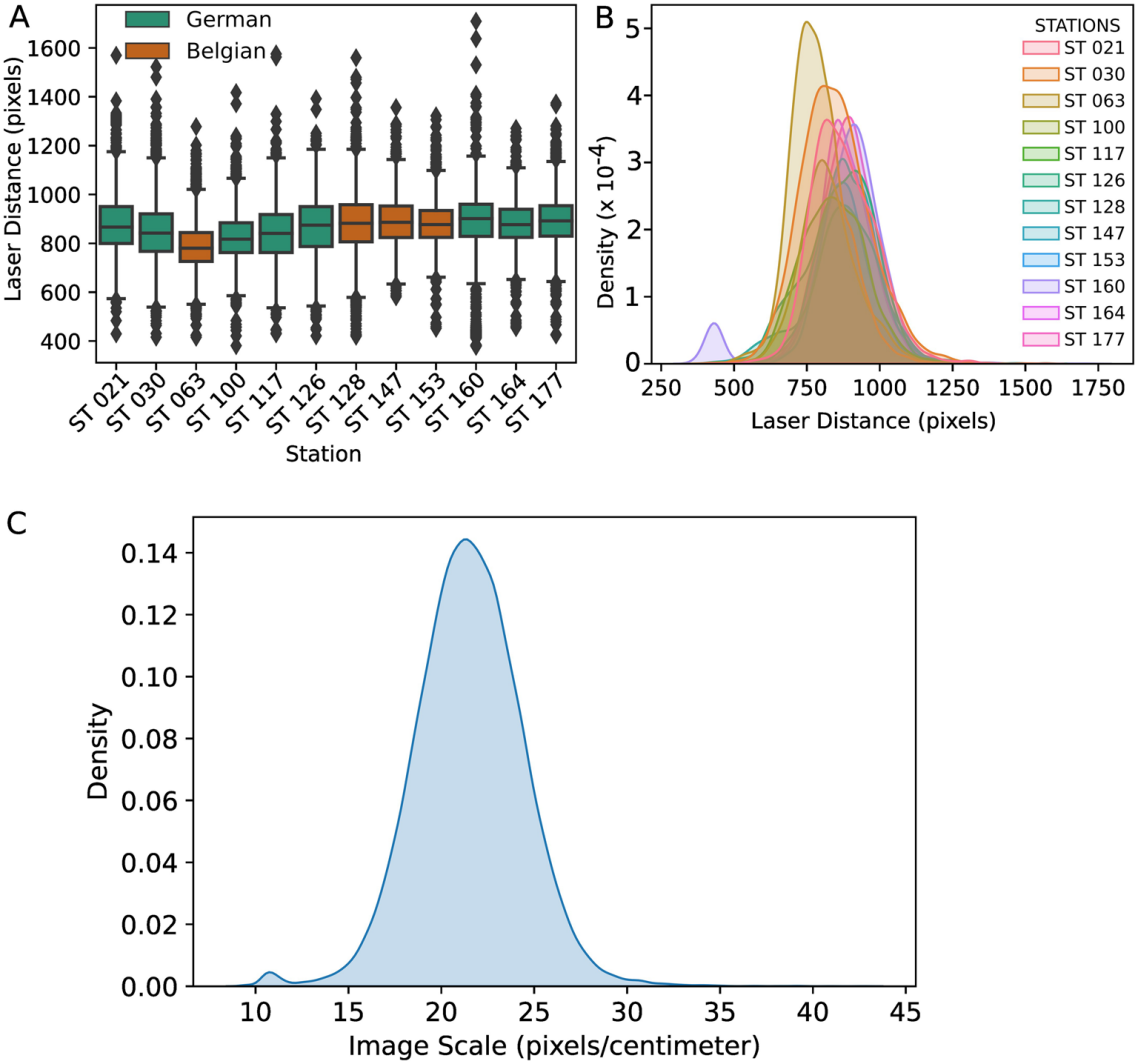
Distribution of detected laser point separation distance in pixels. These are grouped by dive station and represented as (**A**) box plot with whiskers, and (**B**) normalized kernel density estimate. (**C**) Distribution of image scale in pixels/centimeter obtained by scaling the detected laser distance by known distance.

The distribution of the distance in pixels between detected laser points is shown as a box plot and kernel density estimate in Fig. 1A,B. The distributions are given for each OFOS deployment. The average laser separation distance across all images was 860 pixels. The deployment at station 063 contained more than 50% of images with laser distance below the average, which made them visually darker since they were recorded from a higher altitude. The distribution of all image scales in pixels/centimeter (px/cm) is shown in Fig. 1C, and shows a gaussian distribution centered around the mean scale of 22 px/cm.

#### Illumination drop-off correction and contrast enhancement.

**Figure 2:**
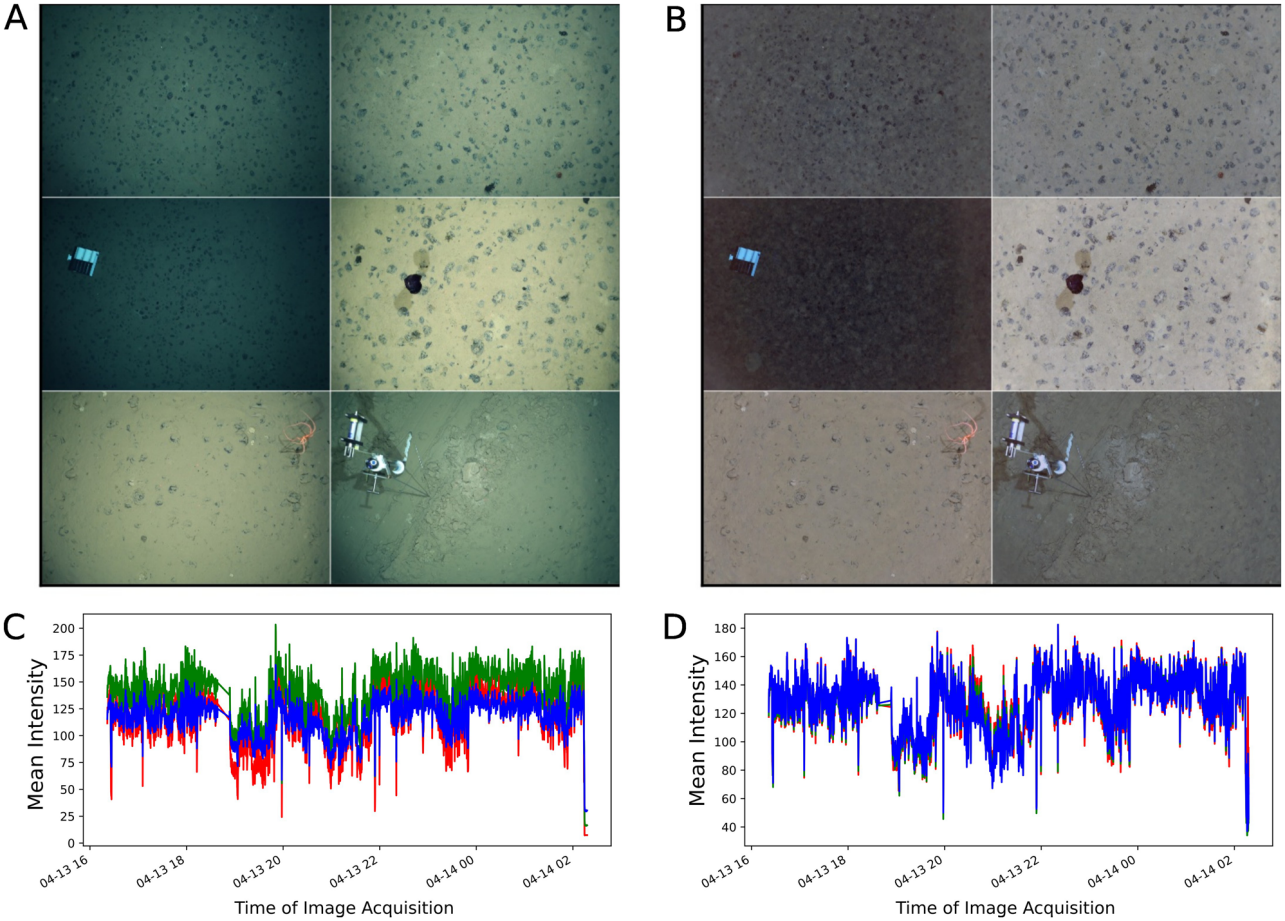
Comparison between sets of (**A**) original images and (**B**) images corrected for illumination drop-off. (**C**) Mean intensity plots calculated for each R, G, B channel in original images and, (**D**) corrected images from OFOS dive 126. Note the altered scale on the y axis between (**C**) and (**D**).

Illumination drop-off caused the edges of the original images to appear darker than their center regions (Fig. 2A). Applying the z-score normalization resulted in uniform scene illumination that reduced the dark image edges (Fig. 2B). The z-score normalization of images follows the gray world assumption, in which neutral gray is assumed to be the average reflected color of a scene with a good distribution of colors^28^. The effect of this was that corrected images exhibit a light olive to brownish appearance. A plot of the mean intensity for each RGB channel is shown for both the original images (Fig. 2C) and the corrected images (Fig. 2D) from OFOS dive 126. Compared to the original images, the mean intensity curves generated from the light drop-off corrected images show strong synchronization.

**Figure 3:**
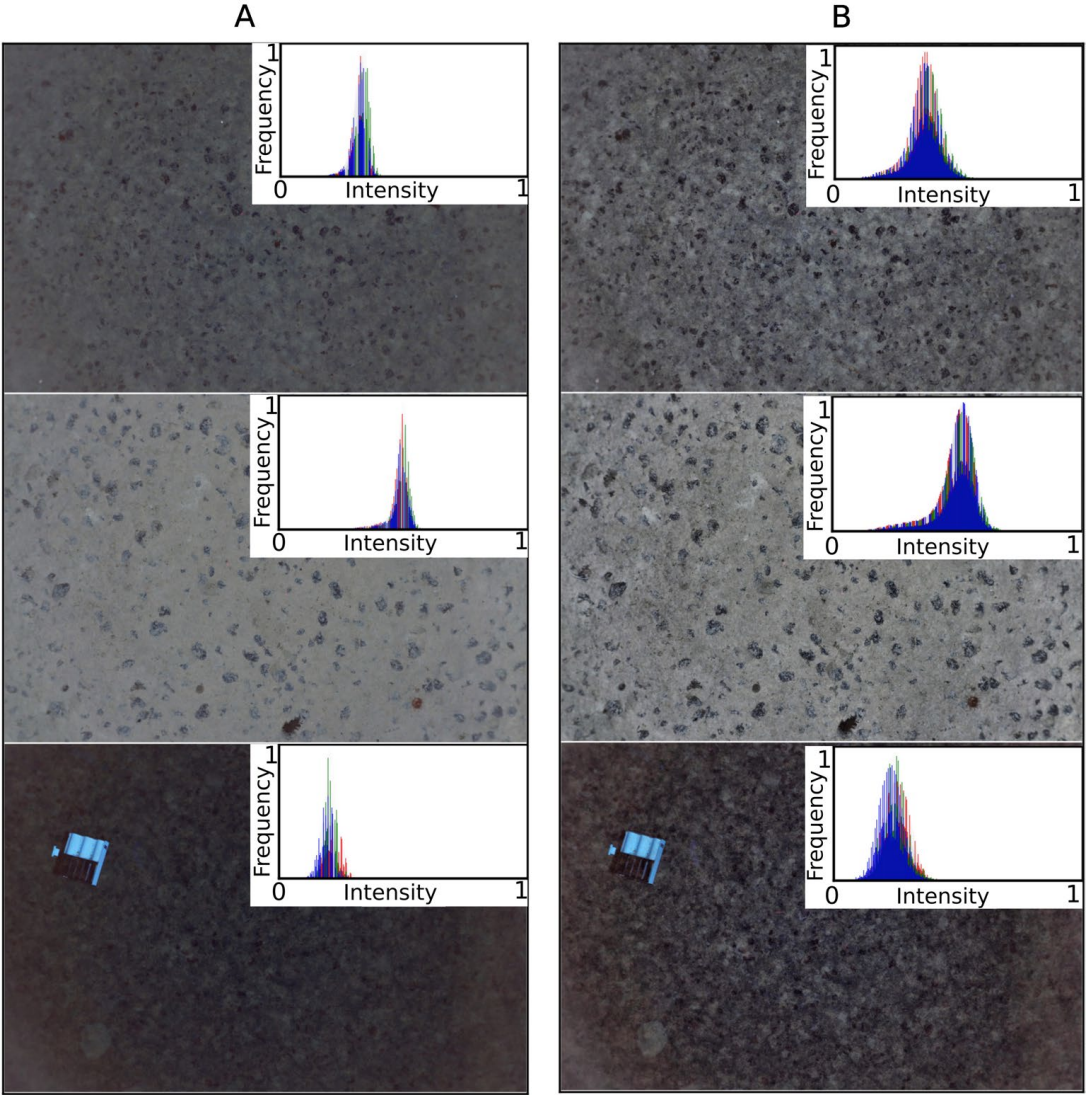
Comparison between sets of (**A**) images corrected for illumination drop-off and, (**B**) adaptive histogram equalized images. Also shown as insets are the intensity histograms showing the distribution of the pixel values for each RGB channel.

The contrast enhancement transformation applied to the light drop-off corrected images qualitatively revealed more visual detail. Figure 3A shows examples of light drop-off corrected images with their corresponding color histograms as insets, whereas Fig. 3B shows the same images after the contrast enhancement transformation. Qualitatively, the intensity histograms of the light drop-off corrected images occupy a narrow range of intensity values. In contrast, the intensity histograms of contrast enhanced images are spread out to utilize the full range of available intensity values. Quantitatively, the entropies of the intensity histograms for the contrast enhanced photos are by a factor of 2 greater than those for the light cone corrected photos. This implies that the pixel values of the contrast enhanced images have a more uniform distribution, which improves their global contrast, and likely makes the images easier to interpret.

#### Color normalization

**Figure 4:**
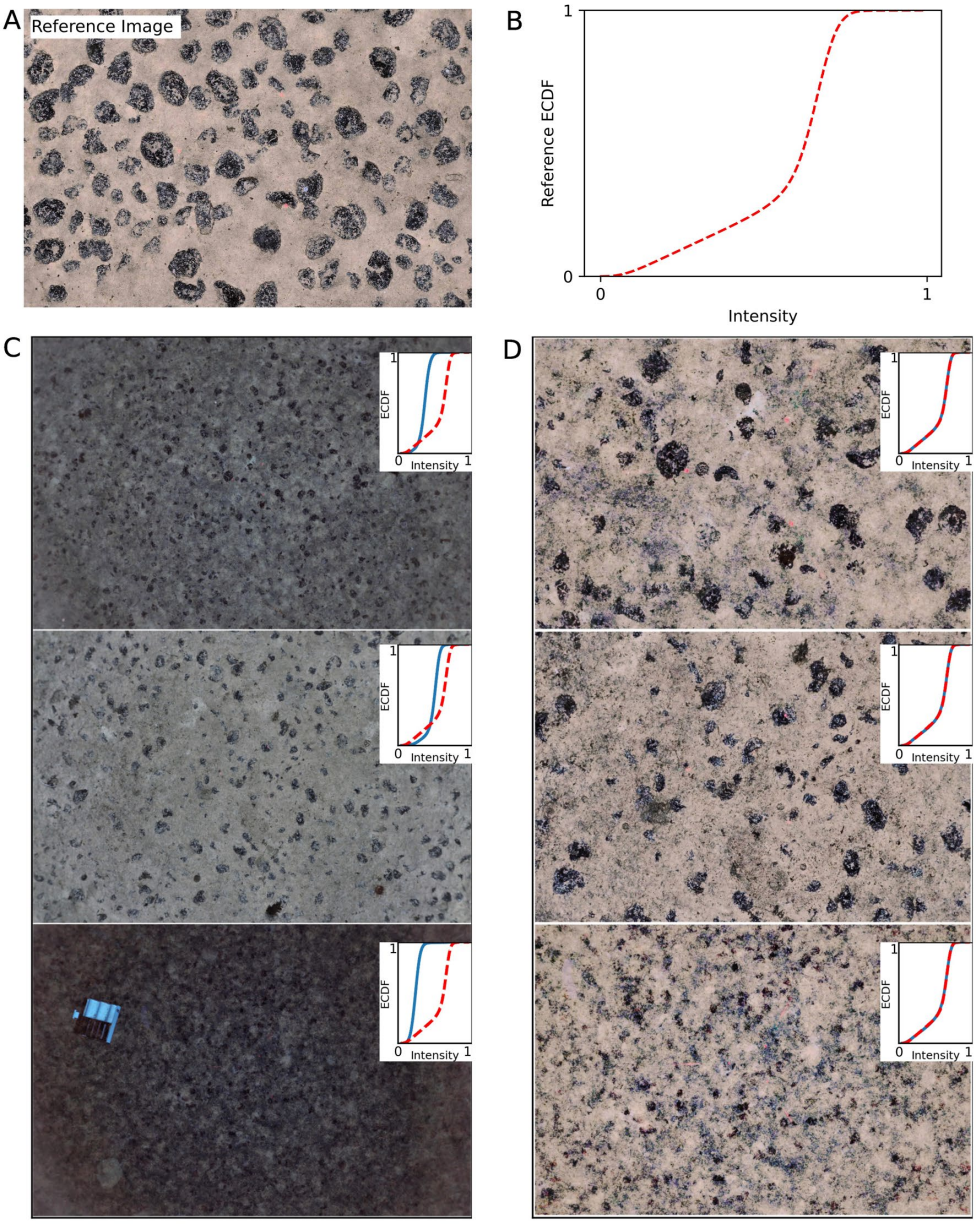
(**A**) Reference image used for color normalization and, (**B**) The Empirical Cumulative Distribution Function for the reference image. (**C**) Comparison between adaptive histogram equalized images and, (**D**) color normalized and rescaled center cropped images.

Figure 4A shows the reference image used for the color normalization; it was chosen as the image that was acquired closest to the seafloor, and therefore it also has the maximum resolution in pixels/centimeter (see details in the supplementary information). The empirical cumulative distribution function (ECDF) for the reference image is shown in Fig. 4B. The color normalization transformation converts the ECDF of all images to match that of the reference image. Figure 4C shows three examples of contrast enhanced images, with their corresponding ECDF drawn as insets. When compared to the reference image, the ECDF for the contrast enhanced photos are shifted left, which indicates that their overall brightness values are low. Figure 4D shows the results of the same images after color normalization by histogram matching the reference ECDF; we point out that these color normalized images have already been transformed to a consistent spatial resolution (in pixel/cm), and then center cropped to represent a standard spatial footprint of 1.6 square meters on the seabed (see details in “Image enhancements” section). From this figure, the resulting ECDF of the color normalized images are identical to the reference distribution. Qualitatively, this transformation results in normalized photos which have a good overall scene brightness.

### Seafloor classification assessment

This section presents results of the quantitative assessment aimed at evaluating the performance for both the supervised and unsupervised classifiers on the test set images. Also provided are the results of the PCA projection of all classified images onto a two-dimensional feature space, which can be used to visualize how semantically similar images group together in the feature space. For example, whereas images of large sized Mn-nodules group together on the upper region of this feature space, images of densely distributed Mn-nodules group together on the left.

#### Performance evaluation of the fine-tuned Inception V3 classifier

**Figure 5:**
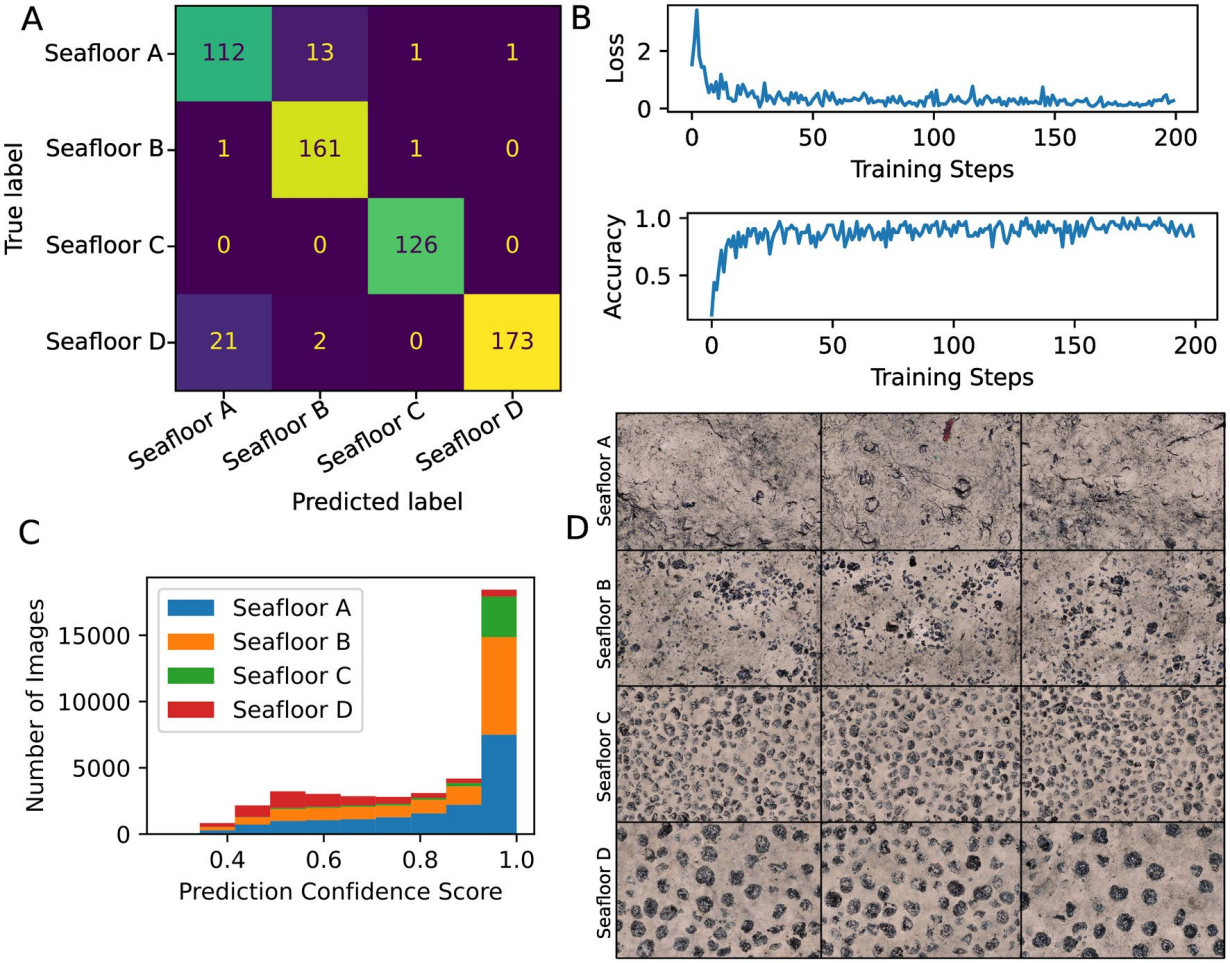
(**A**) Confusion matrix for supervised classifier performance evaluation. (**B**) Loss and accuracy curves used to monitor progress of model training. (**C**) Distribution of prediction confidence scores for each seafloor class. (**D**) Examples of classifier predictions for each seafloor class.

The confusion matrix used to evaluate the performance of the supervised classifier is shown in Fig. 5A. The off-diagonal elements of the matrix tabulate the number of misclassifications made by the supervised classifier when it made predictions on the test images. These test images comprised 12.2% of the labeled images (612 in total), since the other 7% was used as validation set during hyper-parameters tuning. The matrix shows that some of the test images labeled as Seafloor A were wrongly predicted to belong to Seafloor B. This could be because in some images of Seafloor A the sediment blanket cover over the Mn-nodules was not complete (not high enough), and some that were only partly Mn-nodules are still visible; this caused them to be misclassified as Seafloor B. The same reasoning also explains the confusion between Seafloor A and Seafloor D, depending on the general distribution of Mn-nodules (small patches as in Seafloor B or homogenous distribution of large Mn-nodules (Seafloor D).

Figure 5B shows the development of the learning curves used to track the progress of model training after every iteration step. The shape of the cross-entropy loss curve decreases with every iteration, and converges with a final loss value of 0.09. On the other hand, the shape of the accuracy curve rises steadily with each iteration until model convergence.

F1 score was used as the accuracy metric to evaluate the performance of the fine-tuned Inception V3 classifier; it provides the harmonic mean of precision and recall, and ranges between a worst score of 0 to the best score value of 1. Using this approach, the F1 score of our fine-tuned classifier was found to be 0.93.

Figure 5C shows the distribution of prediction confidence scores for each of the seafloor classes. The prediction confidence was greater than 0.6 in more than 80% of the images, for all four classes. Figure 5D shows three example images for each of the four seafloor classes as predicted by the fine-tuned Inception V3 classifier.

#### Performance evaluation of sampling strategies used in fitting k-means classifiers

**Figure 6:**
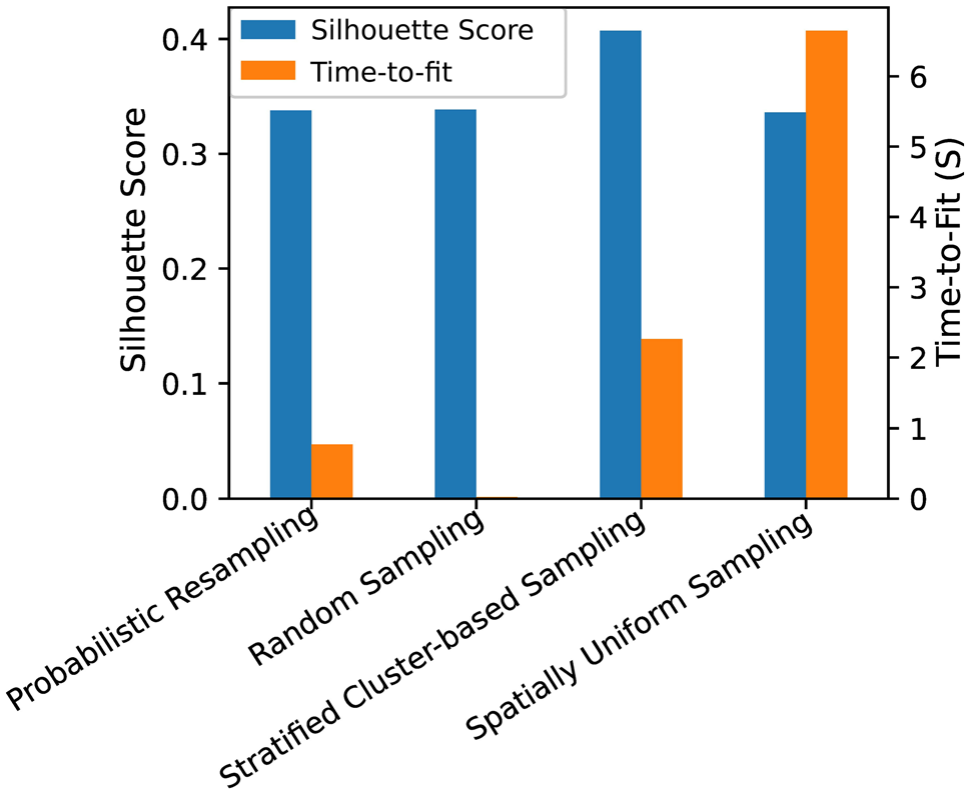
Performance evaluation of unsupervised classifiers fit using training data generated using each sampling strategies.

Figure 6 shows the results of the comparison of the four training set sampling strategies. As mentioned, each strategy was evaluated based on the time it took from generating the training images to fitting a k-means classifier, and also on the quality of the resulting clusters. Random sampling was the quickest with a time-to-fit of 0.12 s. Stratified cluster-based sampling resulted in the best quality of clusters, with an absolute silhouette score of 0.4. Spatially uniform sampling was the slowest with a time-to-fit of 7 s, despite the quality of its clusters being equal to both random sampling and probabilistic weighted resampling. The optimal sampling strategy was chosen as stratified cluster-based sampling. This is because it achieved the highest silhouette score of 0.4, and was only 1.7 s slower than the second fastest strategy of the probabilistic resampling.

Treating the Inception V3 classification results as ground truths, the Fowlkes-Mallows Index (FMI) was used to quantify the success of our k-means classifier in successfully defining clusters that are similar to the ground truth set of classes. The FMI is the geometric mean of the precision and recall that makes no assumption about the cluster structure^29,30^. Using this approach, we obtained an FMI score of 0.5, which indicates good similarity in the classification accuracies.

**Figure 7:**
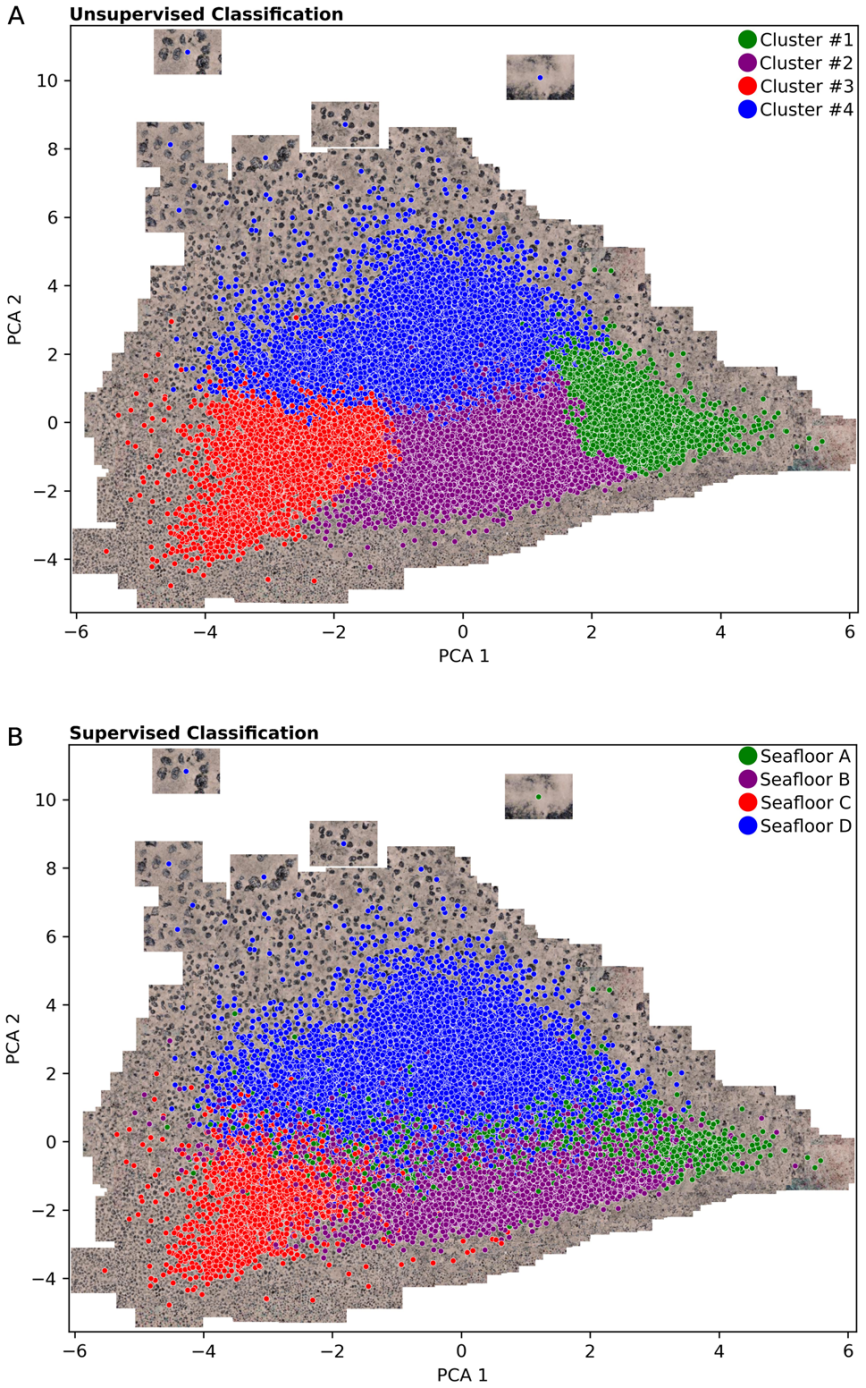
PCA projection of all images onto feature space color coded by results of (**A**) unsupervised classification and, (**B**) supervised classification.

Figure 7 shows the PCA projection of all the images color coded by the results of both unsupervised and supervised classification. The class boundaries of the unsupervised classifier are abrupt and well-defined. On the other hand, the supervised classifier results in fuzzy class boundaries, which is the case in reality since the transition between classes is subtle. Overall, the two classifiers generate results that agree with a Cohen’s kappa coefficient of 0.6.

**Figure 8:**
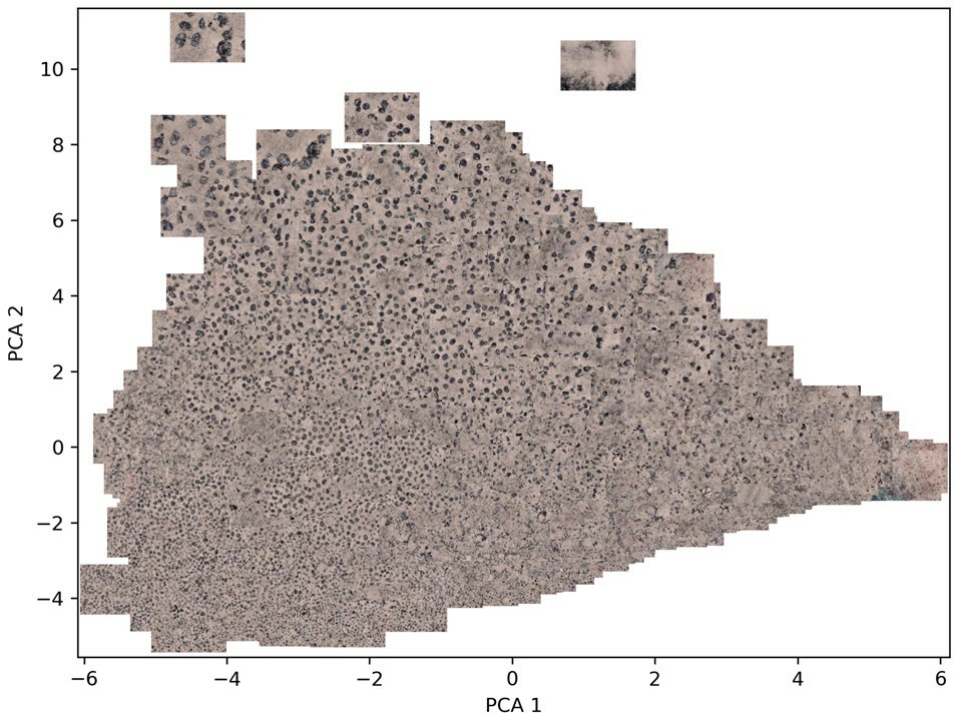
PCA projection of all images onto feature space without color coding. Semantically similar images (e.g., those with large Mn-nodules) can be seen to group together.

Figure 8 shows the PCA projection of all the images without color coding, in which semantically similar images (e.g., those with large Mn-nodules) can be seen to group together in the feature space.

### Spatial distribution of seafloor classes

This section puts the image-based classification results into geographic perspective, by mapping out the distribution of the seafloor classes spatially within both the German and Belgian working areas.

**Figure 9:**
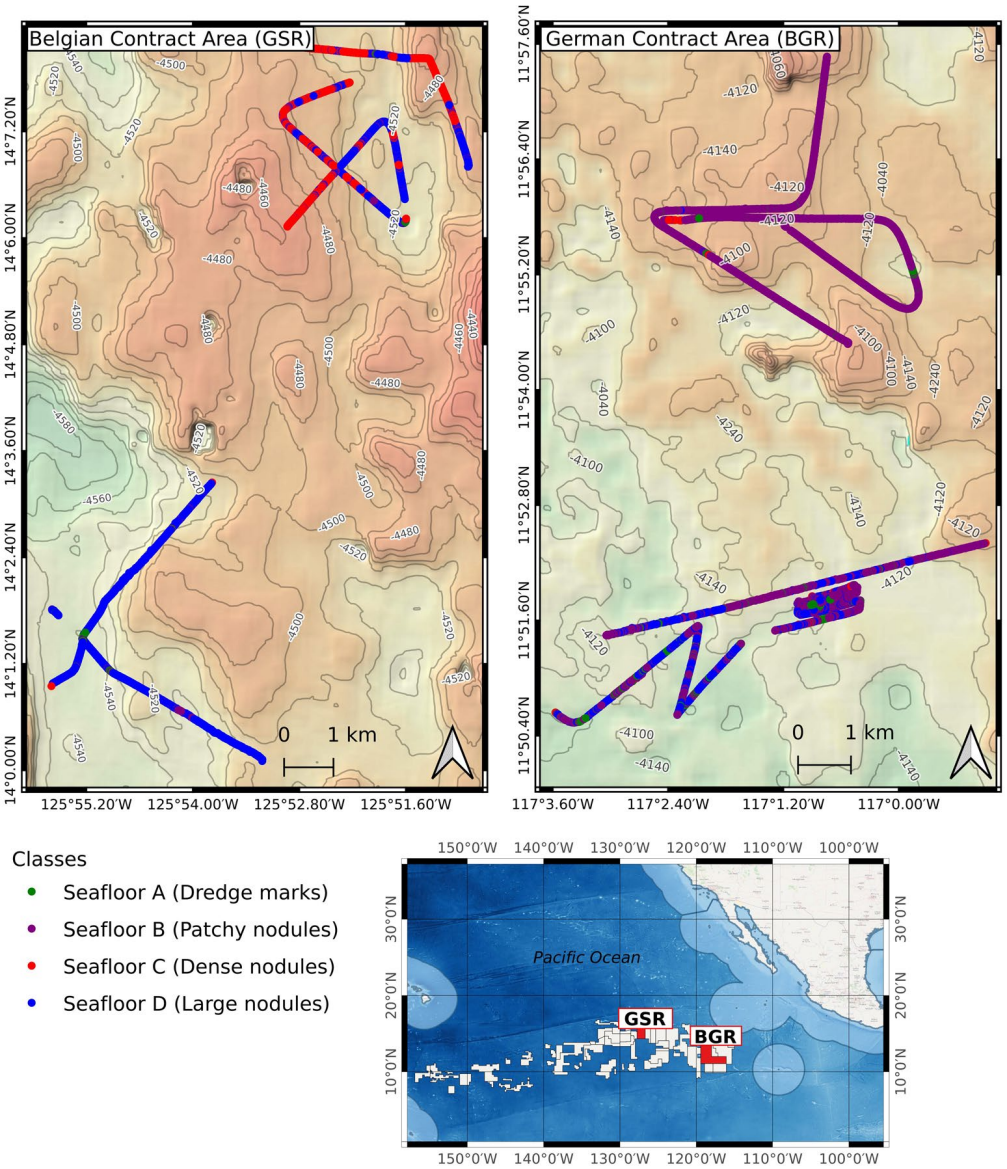
Maps of deployment tracks in both the German (BGR) and Belgian (GSR) contract areas. The deployment tracks are color coded by supervised classification results, and overlaid on our gridded multibeam bathymetric dataset. The boundaries of polymetallic nodule exploration areas were sourced from International Seabed Authority (https://www.isa.org.jm), while the base map in the overview map was sourced from GEBCO (https://www.gebco.net/).

The spatial distribution of the seafloor classes for both German and Belgian contract areas is shown in Fig. 9, by color-coding the image locations along the deployment tracks based on the supervised Inception V3 classification results. The northern half of the Belgian area comprises dense nodule covered seafloor (Seafloor C), intermingled with patches of larger nodules of Seafloor D at local elevations. Seafloor D becomes the dominant class in the depressions in the northern part of the area, and along the two tracks in the southern part. Very few instances of Seafloor class A and B exist in the Belgian area.

**Figure 10:**
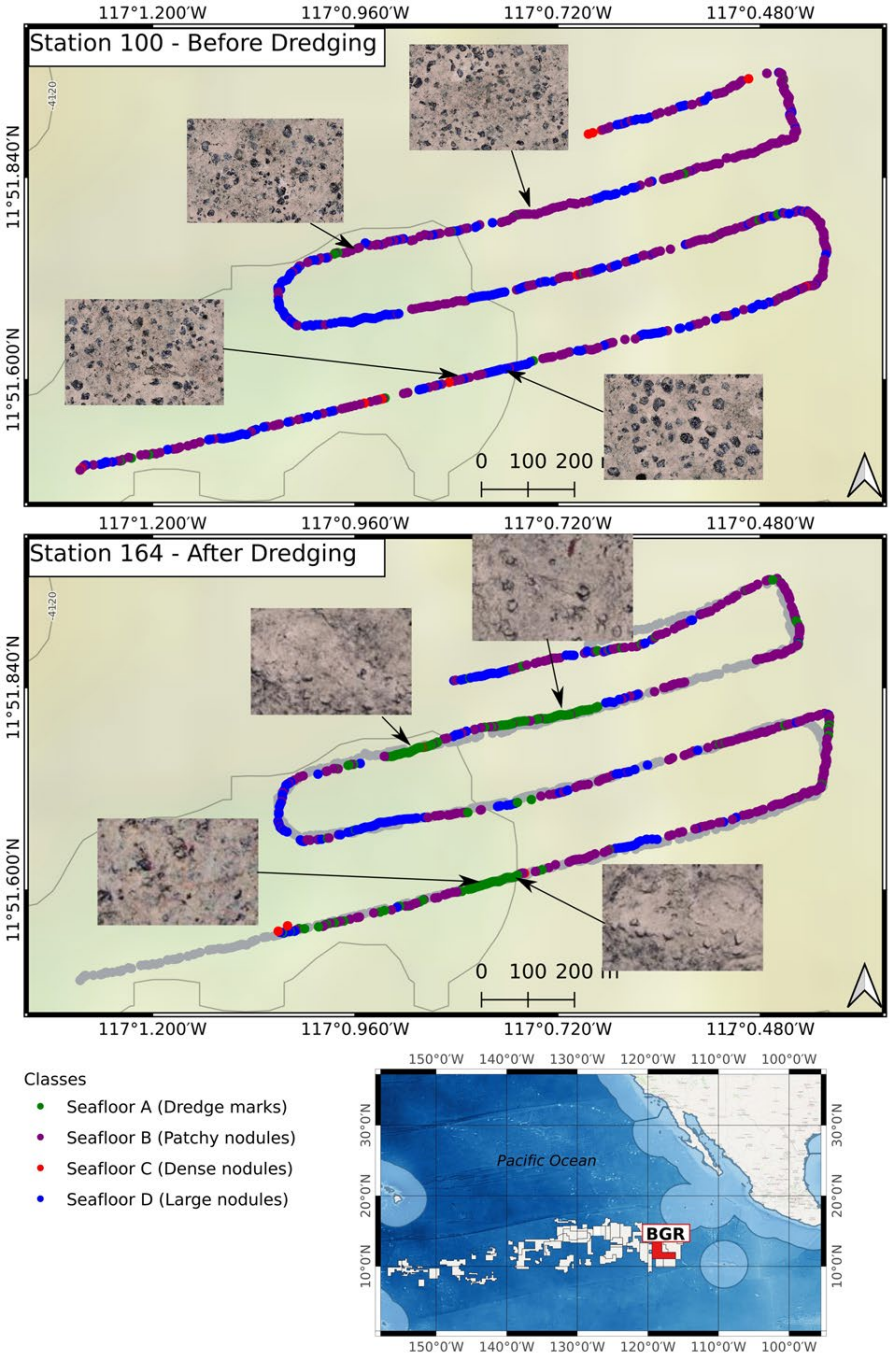
Deployment tracks in the German contract area (BGR), which show the seafloor classification results before and after the dredge experiments. The dredge experiment increased the proportion of sediment-covered seabed (Seafloor A) by 30%. For context, the ‘before’ track is shown in gray overlaid as background in the bottom panel. For clarity, only one observation track is shown from after the dredge experiment.

The German contract area typically comprises Seafloor B in the northern part, and a mix of Seafloor B and D in the southern part. The German area does not contain a significant amount of Seafloor C, highlighting that the Mn-nodules are generally smaller compared to the Belgian area. The occurrences of instances of Seafloor A are clearly correlated to the locations of the dredge experiment conducted in the German area. In Fig. 10, pre- and post-dredge deployment tracks are shown, along with example images. After the dredge experiment, the proportion of Seafloor A increased by over 30%, caused by the sediment turnover/ploughing and subsequent settling of the suspended sediment onto Mn-nodules after a certain time (see also^31^). In order to allow easy visualization of the impact of the dredge experiment, only the post-dredge track that has a corresponding pre-dredge track is shown in Fig. 10. The other two post-dredge tracks also fit with the occurrences from Seafloor A.

## Discussion

For structuring the discussion for clarity, we briefly summarize what has been presented above, and make relevant connections to various aspects. In general, it can be said that recent developments in both underwater imaging technologies and hydro-acoustics have allowed marine researchers to characterize and ground-truth seabed substrate classes^32–34^. In this paper, we present an Automated and Integrated Seafloor Classification Workflow (AI-SCW), which includes a module that automatically detects laser points from images, and uses them for scale determination. This is then used to correct for illumination artefacts, and to color normalize images recorded at different times during different camera deployments. This generates a visually homogenous image data set that can be used as training datasets for the automated classification. To this end, the workflow also includes a module for semi-automatic labeling, which reduces the manual effort required to annotate images needed for training classification models. Both supervised Inception V3 and unsupervised k-means classifiers have been trained, and their classification results compared. The Inception V3 classifier is trained using semi-automatically generated labels, while the k-means classifier is trained using a subset of unlabeled images (those have been generated using a stratified cluster-based sampling strategy). When the results of both the Inception V3 and k-means classifiers were compared, they showed a good agreement, with a Cohen’s kappa coefficient of 0.6. Below, the various aspects of AI-SCW workflow are first discussed in detail. Finally, we discuss the spatial distribution of the derived seafloor classes in the context of terrain and backscatter properties of the seafloor.

As part of the laser point detection workflow, the subtraction of the red channel from the linear combination of blue and green channels worked very well, producing an intermediate image with very high values around the laser points and very low values elsewhere; the intensity maxima of this intermediate image contained the three lasers. We observed that the linear combination had to be scaled by a coefficient to reduce the effect of the correlation among the RGB channels. After some iterations, we found that coefficient values between 0 and 1 produced good results (true positives), and in particular, a coefficient value of 0.2 produced the best results for this dataset. This value may differ when the workflow is applied in other datasets e.g., depending on the lighting condition, depth, type of device recording the images etc. We further observed that detections based on the contrast enhanced images resulted in many false laser spot detections. This is because the contrast enhancement transformation reduced the intensity of the red laser points, by adaptively equalizing the distribution of intensity values in local image tiles. The laser point detection accuracy increased significantly when the detection was done on the original photos. This is because the lasers are targeted towards the center of the field of view of the camera, a region that was already well illuminated by the artificial light source; which made the red laser points prominent and easy to detect. In addition, since the mask used to filter the intensity maxima coordinates was the same for all images, it allowed us to implement the laser point detection workflow concurrently across all CPU cores. This increased the processing speed by up to 3 times, compared to a sequential implementation. When compared to the DELPHI system implemented in a previous study^5^, our implementation relies on manually hand labeled information from just one single example image, compared to the DELPHI system that iteratively uses 70 manually annotated images. As our dataset specifically comprised three laser points projected from a camera altitude of between 1 to 4 m, future research could improve this laser point detection workflow to accommodate other possible scenarios e.g., where there are fewer than three laser points visible in the image.

Our semi-automated approach for generating the labeled training set to be used for fine tuning the supervised Inception V3 classifier was both straight forward and quick to execute. The semi-automation involved some manual annotation of example images by a human analyst, followed by an automated nearest neighbor sampling of additional training images in the feature space. This set-up was used to explicitly include domain knowledge during the labeled training data generation process. Incorporation of domain knowledge was important because nearest neighbor sampling only makes sense when semantically similar images are mapped close together in feature space. In our case, domain knowledge was incorporated by encoding texture and entropy into the feature vectors that represent the images in feature space, because the seabed in our working area was mostly characterized by Mn-nodules that appear as irregular blobs of black pixels in the image. The use of texture features to encode seabed characteristics has been reported in previous studies, which found that they adequately reveal subtle variation in seabed characteristics^19–21^. In addition, domain knowledge was incorporated by interactively choosing example images that are uniformly distributed in the two-dimensional PCA-projected feature space; this reduced the class imbalance in the sample training set. We emphasize that the above-described feature space was only used for generating labeled training data, after which deep learning was employed for supervised classification. Focusing on supervised classification, we observed that the Inception V3 model, which was fine-tuned using the semi-automatically generated labeled dataset achieved a good classification performance. This can be attributed to the ability of deep learning architectures to extract useful abstract features during training, as pointed out e.g. by^35^. Achieving such a good performance using a handful of human-labeled examples partly addresses the bottleneck of manual annotation of large data sets. Therefore, our approach contributes towards making the adoption of state-of-the art computer vision models into image based marine studies much easier.

When exploring the results of the unsupervised classification in the PCA-projected feature space, we observed that the inter-cluster separation distance was small. This observation is typical for images of the deep-sea, where the variation in seabed characteristics is subtle over kilometer scale, as was also pointed out in previous studies such as^36^. Our comparison of the four strategies for selecting the appropriate training data for fitting an unsupervised classifier showed that with such images, random sampling may be the ideal approach for generating this training data set, if quick computation is most important. Despite the speed, however, random sampling may cause imbalance in the training data, since samples will be disproportionately drawn from regions of the feature space that are over-represented. This is likely to occur e.g., when images are collected in the largely homogenous abyssal Mn-nodule plains with only infrequent regions of no (sediment covered) nodules or other rare seabed characteristics. In a previous study, Prati et al.^37^ also corroborate this relationship between random sampling and class imbalance. Furthermore, training a classifier using imbalanced data may result in poor generalization performance, as was also pointed out by^38^. When the metric of interest is not speed but quality of the resulting clusters, our experiment showed that stratified cluster-based sampling is a better approach. This becomes evident as it resulted in the highest absolute silhouette score, while being only 2 s slower than random sampling. The high silhouette score implies that of all the other methods we compared, the stratified cluster-based sampling approach resulted in clusters that are clearly distinguished from each other. This is because the approach incorporates an over-clustering step that first partitions the entire entropy-defined feature space, which allows samples to be drawn uniformly across all regions of the feature space, naturally reducing class imbalance. Data from a more heterogenous seafloor like in shallow water environments may not exhibit a similar feature space distribution, and therefore a different sampling strategy may be required that needs to be tested in future studies.

Visualizing the unsupervised seafloor classification results of the k-means clustering in feature space showed abrupt inter-class boundaries. This is an artifact of the k-means objective function that results in convex clusters with well-defined boundaries, as was also pointed out in a previous study by^39^. In the literature, there have been attempts to explicitly introduce fuzzy class boundaries in seafloor classification e.g.^40^. Others such as^41^ attempted to customize k-means by modifying its objective function using least-squares criterion to implicitly introduce fuzziness. However, the authors clearly point out that these fuzzy clustering approaches are very sensitive to cluster size imbalance, and can result in clusters whose semantic meaning is hard to interpret. In our supervised case of the Inception V3, the boundaries were logically fuzzy in the PCA-projected space because the convolutional neural network outputs a set of probabilities. This allows the model to make soft classification by assigning each image a likelihood of belonging to each of the classes using a confidence score. Despite these general differences, our quantitative assessment of both the supervised Inception V3 and unsupervised k-means classification results indicate a good agreement, with a Cohen’s kappa coefficient of 0.6. McHugh^42^ also quantified the extent to which two different classifiers assign the same score to a variable using the Cohen’s kappa statistic. From these assessments, it can be concluded that a fast, preliminary seafloor classification while at sea may be performed with unsupervised methods. The main advantage of using k-means is the low computing power needed, which nevertheless produces reasonable classification results. Supervised methods which require GPU computing resources may be better executed for seafloor classifications as part of a detailed habitat characterization in a second step. They are better suited to pick out subtle transitions between classes, which is usually the case in reality.

Regarding the geospatial distribution of seafloor classes and their correlation with bathymetry, the visualization of our seafloor classes in map view makes it obvious that their spatial distribution is not random/homogenous but clustered. In the Belgian area, the dense Mn-nodules (Seafloor C) and large sized Mn-nodules (Seafloor D) occupy 94% of the OFOS-inspected area at about 4500 m water depth. The German contract area pre-dominantly contains sparsely distributed Mn-nodules (Seafloor B) in the North, while the south comprises a mixture of both Seafloor B and Seafloor D. Overall, 96% of sparsely distributed Mn-nodules make up the seafloor in the German area at water depths of approximately 4100 m. The seafloor class D with big Mn-nodules lies in deeper regions of the seafloor with rugged terrain, whereas sparsely distributed nodules occupy shallower regions of the seafloor with flat terrain (please see further details in the supplementary information). This is consistent with the findings of prior studies, which showed that a higher number of Mn-nodules occur in areas with a rugged seafloor than flat plains e.g.,^41,43,44^. At the site of the dredge experiment, stretches of sediment covered, or dredged/ploughed seabed (Seafloor A) appears after the experiment; the proportion of sediment covered seabed increased by 30% (see Fig. 10).

Comparing our approaches to related works, our color normalization approach based on automatically detected laser points introduced a simple yet novel workflow, which significantly reduced the underwater illumination artifacts on our images. Similar to previous works e.g. by^24^, our approach rescaled and normalized the color of each image depending on its altitude above the seafloor: images recorded farther from the seafloor were transformed more than those close to the seafloor. In contrast, however, our approach was different since we did not have access to the altitude of each image, and therefore we implemented a novel approach that infers them from automatically detected laser points. Furthermore, our approach uses histogram matching to correct for uneven scene brightness among the photos, which is simple, parallelizable, and does not even require knowledge of parameters required for reconstruction of the path of light rays through the water column e.g. as demanded by physics-based approaches^24,45,46^, or photogrammetric structure from-motion^47^ and simultaneous localization and mapping (SLAM)^48,49^. Moreover, performing color normalization on the raw images improved the accuracy of seafloor classification; this is similar to observations by previous works such as by^24,45^. Particularly in our case, the raw images acquired at varying altitude were not directly comparable; these images represent regions of the seafloor with varying spatial foot print and scene brightness. With respect to generating annotations for training classifier, our semi-automated labeling approach greatly reduces human effort similar to previous works by^11,50,51^. However, our approach is novel because it allows for the incorporation of domain knowledge through feature space engineering, rather than only relying on similarity in geographic space and spatial auto correlation e.g. as proposed by^52^. Regarding optical image-based seafloor classification, our results revealed seabed substrate classes that had semantic meaning, similar to previous works by^20,21,24,25,53,54^. However, our workflow is novel since we implement both supervised (Inception V3) and unsupervised (k-means) classifiers, and we further demonstrate that both of them show a good overall agreement in seafloor classification accuracy. Thus, our study provides an unsupervised seafloor classification workflow that can be used at sea where computers are not very powerful, as well a supervised workflow that is suitable for office settings where there is access to computers with increased memory and GPU hardware.

## Conclusion and recommendations for future research

This study contributes to the current understanding of image-based deep seabed classification, and its novelty can be summarized as follows: First, we implement a new approach that automatically detects laser points from a sequence of underwater images. This is useful for calculating scale, which allows marine researchers who make measurements on the images to convert from pixel units to real world metric units e.g., meters. The scale also allows the scientists to infer the height above the seafloor from which the image was acquired, which is useful for determining the spatial footprint of the image; Second, we implement a semi-automated approach that drastically reduces the required human effort during image labeling. This is useful for marine scientists who routinely work with large volumes of underwater images, since it reduces the burden and fatigue of generating manual annotations, while still allowing them to train high-accuracy classification models (e.g., convolutional neural networks, random forests etc.) that automatically analyze the images and subsequently classify the seafloor into habitat types; Third, we propose stratified cluster-based sampling as good strategy for generating a subset of images to be used for training an unsupervised classifier. When faced with huge volumes of images following an expedition, this proposed sampling strategy is useful to help marine scientists when deciding how to generate a training set that fits in standard computer memory, while simultaneously being class balanced and not requiring specialized hardware such as GPU.

Although we propose stratified cluster-based sampling as the optimal unsupervised classifier, this conclusion is specific to underwater photos recorded in the deep-sea abyssal plains, for which the seafloor is mostly homogenous. This may not hold in shallower depths with heterogenous seafloor characteristics, and future research could address this by extending our AI-SCW feature extraction module to investigate e.g., if color differences could be useful in classifying seafloor covered by other substrate classes such as seagrass, rocks and sand. In addition, future research could explore the possibility of embedding automated image-based workflows such as AI-SCW onto the data acquisition software of ROV/UAV. This would enable real time on-the-fly image analysis, which provides significant savings in time and post processing effort for applications such as seabed classification, megabenthic fauna detection as well as the quantification of crusts and nodules; these are ongoing developments at GEOMAR and other centers.

As a concluding remark, this study comprehensively presents a set of methods that together form an automated workflow for image-based seafloor classification and mapping. These include: automated laser point detection; semi-automated image annotation; as well as supervised and unsupervised seafloor classification. The applicability of these methods is demonstrated using a case study involving seafloor images from Mn-nodule covered deep seabed areas of the Clarion Clipperton Zone in the Central Pacific Ocean. In so doing, we clearly demonstrate potential ways of incorporating recent advances in machine learning and computer vision into marine research, especially for purposes of generating actionable insights from the huge volumes of optical underwater imagery, which are nowadays recorded during scientific expeditions by imaging systems such as AUV, ROV and OFOS. We believe that these insights significantly contribute towards the broader aim of understanding our marine ecosystems, which in turn enables appropriate measures to be established for their management and sustainable use.

## Materials and methods

### Working area

As a case study, AI-SCW was applied to an underwater image dataset recorded during an expedition to the German and Belgian contract areas for manganese nodule (Mn-nodule) mining, at the Clarion Clipperton Zone (CCZ) in the central Pacific Ocean. The expedition was part of the second phase of the JPI-Oceans project MiningImpact, and was executed on board the German research vessel SONNE during cruise SO268. The project aimed at assessing how potential mining of polymetallic nodules on the seafloor would impact the deep-sea environment. A total of 12 video investigations were performed in two different contract areas (Table 1). Within the German contract area, a small-scale sediment plume experiment was conducted, using a chain dredge to observe the re-depositioning of plume sediments before and after the disturbance. Three camera deployments were conducted to photograph the seafloor after the dredge experiment, while one deployment was conducted before the experiment for comparison^55^.

**Table 1:** Overview of the OFOS deployments in the Clarion Clipperton Zone during cruise SO268.

Station	Contract area	Depth (m)	Approx. track length (km)	Approx. bottom time (h)	Number of pictures taken at the seafloor
SO268-1_21-1_OFOS02	German	4538	9.2	11.0	3921
SO268-1_30-1_OFOS03	German	4070	10.7	12.0	4981
SO268-2_100-1_OFOS05	German Dredge (before)	4247	5.0	8.6	2749
SO268-2_117-1_OFOS06	German	4109	7.7	8.5	2956
SO268-2_126-1_OFOS07	German Dredge (after)	4117	8.8	10.0	3492
SO268-2_160-1_OFOS11	German Dredge (after)	4115	11.0	10.0	3526
SO268-2_164-1_OFOS12	German Dredge (after)	4118	5.0	9.0	3075
SO268-2_177-1_OFOS13	German	4127	7.3	9.5	3414
SO268-1_63-1_OFOS04	Belgian	4478	10.5	12.5	4532
SO268-2_128-1_OFOS08	Belgian	4486	5.5	8.0	2749
SO268-2_147-1_OFOS09	Belgian	4519	5.8	8.0	2981
SO268-2_153-1_OFOS10	Belgian	4522	6.0	8.0	2302

#### Setup of the image acquisition system

The underwater images were acquired using an Ocean Floor Observation System (OFOS), which was towed at a speed of 0.5 knots at 1 to 4 m above the seafloor. It comprised a steel frame equipped with both still and video cameras. The still images were recorded using a Canon EOS 5D Mark IV camera with a 24 mm lens, whereas video was recorded using the HD-SDI camera with 64° × 40° view. These two cameras were spaced 50 cm apart and directed vertically towards the seafloor alongside two strobe lights (Sea&Sea YS-250), four LED lights (SeaLite Sphere), three lasers spaced 40 cm apart, one altimeter and one USBL system for tracking the position of the OFOS. Whereas the video camera recorded continuously, the still camera took an image once every 10 s. Both cameras had a dome port that did not alter the field of view as long as the lens was centered properly within the dome. Camera calibration was done by photographing a camera calibration target on deck before the actual deployment. Further details regarding the image acquisition set up can be found on page 65 of the SO268 cruise report^55^.

#### Image dataset

The image dataset comprised 40,678 underwater still images, which were recorded during the 12 deployments of the towed Ocean Floor Observation System (OFOS). The respective data are published on PANGAEA^56^, and can be accessed online (https://doi.pangaea.de/10.1594/PANGAEA.935856). In addition to the PANGAEA dataset, the images can also be accessed upon request as services through the BIIGLE portal (https://annotate.geomar.de/projects/44). BIIGLE is an online image annotation platform, specifically developed to facilitate the annotation of benthic fauna from underwater images^57^. The OFOS deployments were conducted in an average water depth of 4,280 m, covering a track length of 92.5 km in total. After acquisition, the images were georeferenced by matching each image’s acquisition time in UTC to the USBL navigation data. Three laser pointers in a triangular configuration are used for scaling. Light is provided through several lights focusing on the central area below the OFOS frame illuminating the field of view of the vertically downward looking camera. Detailed information about these images and their acquisition can be found in the SO268 cruise report^55^. Table 1 provides an overview of the OFOS deployments during the expedition.

### Software and APIs

AI-SCW has been implemented using the Python programming language. Some of the major libraries used include scikit-image, scikit-learn, TensorFlow and pandas. Supplementary Table S1 provides a complete list of all the specific python libraries used in AI-SCW as well as a brief description of their use. In addition, the specific python scripts used in implementing each component of AI-SCW is shown in supplementary Table S2. All of these scripts can be found online in this public Gitlab repository (https://git.geomar.de/open-source/AI-SCW), where the complete source code files for the AI-SCW project are open source. Alongside the source code files, a detailed guide for setting up the programming environment and executing the respective scripts for each component of AI-SCW is provided.

### Image enhancements

#### Scale determination by laser point detection

Three laser points projected onto the seafloor and photographed in each image are used to determine the image scale. This scale is useful for marine scientists who rely on measurements made on the images, since it forms the basis for the conversion from pixel units to real world units. The scale is calculated as a ratio between the distance separating the three laser points (in pixel units) and their calibrated distance measured in real world units (centimeters). Manually annotating the laser points from thousands of images is laborious and non-scalable. This provides the primary motivation for automating the laser point detection. Below, we describe an approach for automatically detecting lasers from each image, and using these detections to calculate the scale for each corresponding image.

Each image *I* of the data set consists of three-color channels (*I*^*(R)*^, *I*^*(G)*^, *I*^*(B)*^*)* and each of these channels has a pixel width *w* = *4480* and pixel height *h* = *6720*. One training image with well visible laser points is manually selected and annotated. The annotations provide an estimate for the pixel coordinates of laser points in all images. This is done by creating a mask *M*_*lp*_ as triangle which connects the three annotated laser points. To allow for variability in laser point coordinates caused by varying OFOS altitude, a buffer is added around *M*_*lp*_. This buffer is chosen as 250 pixels in our implementation. It was determined by randomly checking different images of varying altitudes to evaluate if laser points indeed fall within the buffered mask. The three annotated laser points provide the average pair-wise distance *d*_*lp*_ between laser points in pixel units.

To detect the red laser points in an image *I*, first a linear combination of its color channels is used to generate a laser signal image ${I}^{(LS)}$:

1 $${I}^{(LS)}= {I}^{(R)}-0.2({I}^{(B)}+{I}^{(G)})$$

Equation 1 was derived from the observation that since the pixels around the laser points were bright red in color (high values in the red color channel), it follows logically that the blue and green color channels had low values in the same region of the image. Therefore, subtracting a linear combination of blue and green color channels from the red color channel would result in an intermediate image, which has very high values around the laser points and very low values elsewhere.

**Figure 11:**
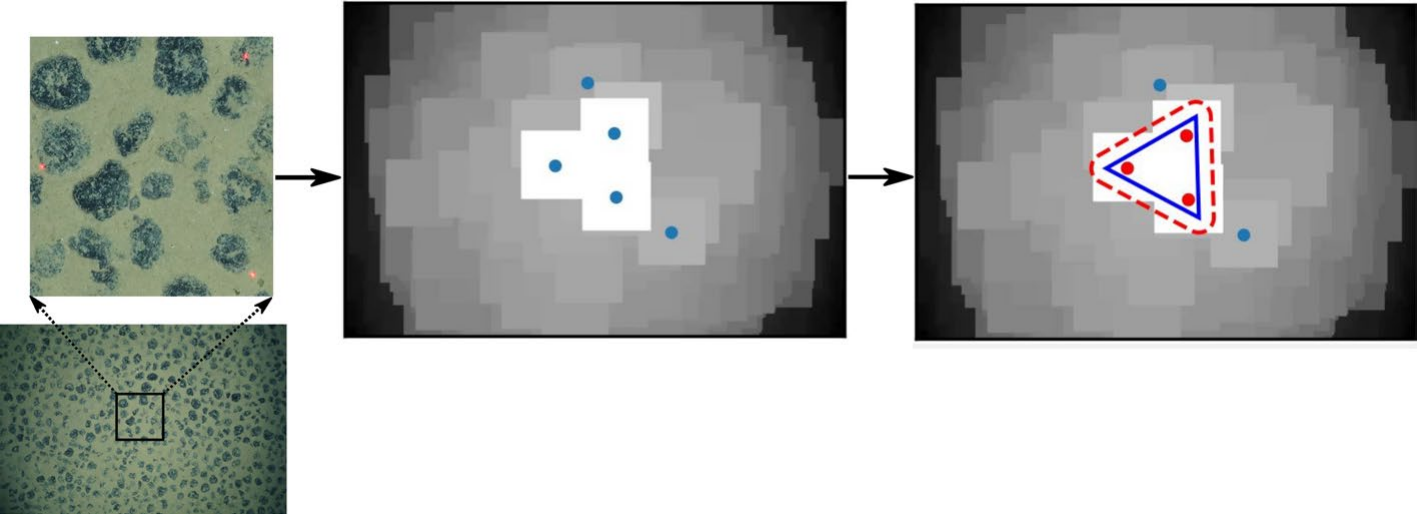
Progression of laser point detection. The local maxima of the laser signal image *I*^*(LS)*^ contain potential laser points (shown in blue). The desired laser point coordinates (shown in red) fall within the buffered mask *M*_*lp*_.

Therefore, laser points of *I* then are among the intensity maxima in *I*^*(LS)*^. These maxima are determined by a local peak-finding algorithm^58^. This algorithm is set to return maxima with a minimum pixel distance of *d*_*lp*_. The laser point coordinates *lp*_*i*_ for *I* are then chosen as those three or less maxima that fall within the mask *M*_*lp*_. An example of the subsequent steps of the laser point detection workflow is shown in Fig. 11.

The image scale *s* of *I* is computed by dividing the average of the pair-wise laser point distances *d*_*i*_ (*i* = 0,..,|*lp*|) by the known real-world distance of the laser pointers of 40 cm:

2 $$s=\frac{\sum_{i=1}^{|lp|}{d}_{i}}{|lp|*40 }\left[\frac{px}{cm}\right]$$

#### Illumination and color normalization

**Figure 12:**
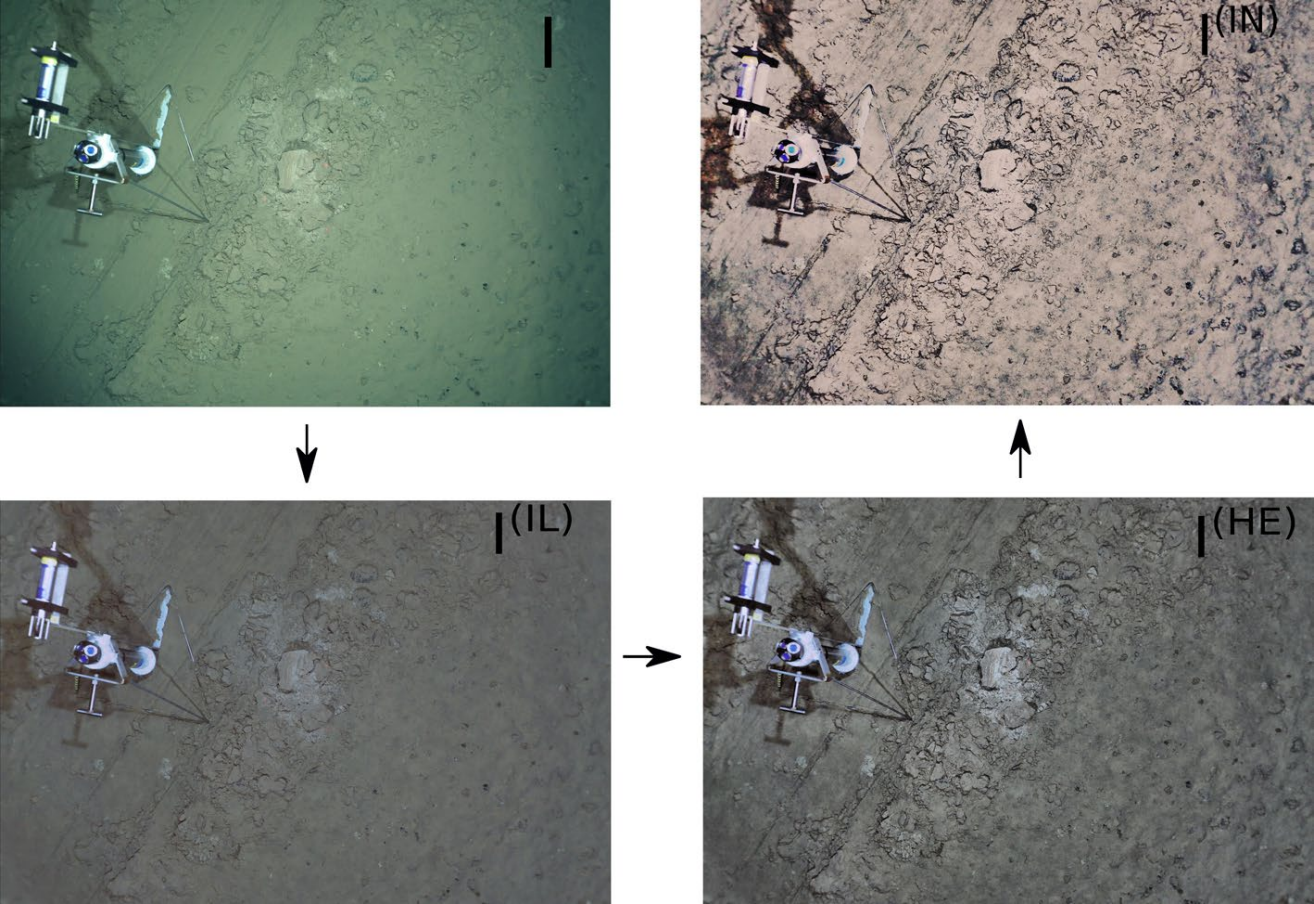
Example showing the color normalization transformations positioned counterclockwise. The transformation progresses from original image *I* to illumination drop-off corrected image *I*^*(IL)*^ to histogram equalized image *I*^*(HE)*^ and finally to the color normalized image *I*^*(IN)*^.

Since tasks such as image-based seafloor classification depend on the visual quality of the images, illumination and color normalization needs to be applied before any automated classification can start. This is necessary to heuristically remove attenuation, scattering and altitude effects caused by the water, the artificial light and the vertical movement of the OFOS. In this paper, illumination and color normalization was done in three processing steps: First, z-score normalization was applied to remove illumination drop-off towards the corners/edges of each image. Second, adaptive histogram equalization was applied to maximize contrast in each image. Finally, histogram matching using a reference image with the maximum resolution was applied to correct for uneven brightness among the images by equalizing their intensity distribution. The detailed mathematical formalisms for each of the three processing steps can be found in the supplementary materials, and also in the user guide in the GitLab repository. An example image processed through each of the three steps of illumination and color normalization is shown in Fig. 12.

#### Standardizing the spatial image footprint

To ensure that each image represented an equal area on the seafloor in square meters, the color normalized images were transformed to a consistent spatial resolution (pixel/cm).

To achieve this, first, the median scale ${Sc}^{M}$ was obtained from the set *Sc* comprising image scales of all the *N* images as:

3 $${Sc}^{M}= \frac{{Sc}_{( \frac{N}{2})}+{Sc}_{\left(\frac{N}{2}\right)+1}}{2}$$

The scaling factor ${F}^{i}$ for an image indexed by *i* was then calculated as the ratio between its image scale ${S}_{i}$ and ${S}^{M}$:

4 $${F}^{i}= \frac{{S}_{i}}{{S}^{M}}$$

This scaling factor was used to resize and resample each image using third order spline interpolation. The rescaled images were then center cropped to a standard size of *width* = *2240* and *height* = *3360,* which corresponds to a spatial footprint of approximately 1.6 m^2^ in each image (here 21.6 pixel/cm).

### Seafloor classification

The automation of image-based seafloor classification is done by training a classifier using a subset of images. During training, the classifier learns a function that maps the training images to their corresponding labels. After training, the classifier is then used in inference mode to predict the seafloor class label for all remaining images. Below, we present both supervised and unsupervised seafloor classifier approaches implemented in AI-SCW using the python scripts indicated in supplementary Table S2.

#### Supervised seafloor classification

##### Semi-automated image labeling

Supervised classification relies on labeled images to train a classifier. However, the generation of these labeled images requires expert annotators to manually inspect a large number of images and assign a label to each image. This is very time consuming and is also not scalable given the large number of images collected during each camera deployment. Therefore, a semi-automated labelling approach was implemented in AI-SCW to reduce the effort required from the human annotator. This was done by first grouping semantically similar images within the feature space. Nearest neighbor sampling was then used to automatically sample images that fall in the neighborhood of manually annotated example images. The feature space was defined by 6-dimensional feature vectors, each of which encoded texture and entropy feature information extracted from the images. The motivation for choosing these features was their ability to describe the Mn-nodule covered seafloor of the CCZ; Mn-nodules appear on the images as irregular blobs of dark pixels. With other images and seafloor characteristics, potentially other features may give better discrimination results, and these features would need to be identified first. Feature extractors such as Resnet-50^59^ that are pre-trained on terrestrial image datasets did not yield a semantically meaningful feature space for our images. This is because Resnet-50 and other models require further fine-tuning using underwater images.

In detail, texture features were generated by converting each image to gray scale, and extracting its connected components. Connected components are pixels with similar intensity that are connected to each other. In each image, the properties of these connected components were measured, aggregated and concatenated to form the texture properties vector. These properties included median size of convex hull; upper quantile of the area in pixels; total area of the components in pixels; density and total count of components. In addition, local entropy^60^ was extracted to reveal the subtle intensity variations within the neighborhood of each pixel. The total entropy was appended to the texture properties vector to form the final 6-dimensional feature vector. A data matrix *X* was then generated by stacking together the feature vectors extracted from all the images as row vectors, after which it was standardized column wise by removing the mean and scaling to unit variance. Linear Kernel Principal Component Analysis^61^ was applied to the this data matrix *X* to linearly project each feature vector to a 2-dimensional feature space for visualization purposes. The human analyst inspected this feature space, and chose 50 uniformly distributed example images for hand labeling. The set of labeled images *T*^*(labeled)*^ was then automatically generated by sampling 100 nearest neighbors around each hand labeled image. The sampling size was chosen to ensure that the set of labeled images was at least 10% of the total image dataset. In particular for this study, the 5000 sampled images corresponded to 12.5% of the entire image dataset comprising 40,211 images. This semi-automated labeling was only possible because the feature space mapped similar images close to each other.

Using this workflow, it was possible to label each image as belonging to one of four seafloor classes (see Fig. 5D). Class *Seafloor A* comprised images showing seabed with no or only few Mn-nodules; this class also contains seafloor dredge marks and turned-over sediment similar to ploughing impacts; Class *Seafloor B* comprised Mn-nodule patches that partly cover the seabed; class *Seafloor C* comprised Mn-nodules densely distributed per unit area; and class *Seafloor D* comprised Mn-nodules that were qualitatively large sized relative to those in classes *Seafloor B* and *C*.

##### Fine-tuning inception V3 supervised classification

A training set comprising 80% of the labeled images *T*^*(labeled)*^ was used to fine-tune an instance of the Inception V3 classifier^27^. This classifier is a convolutional neural network pre-trained on ImageNet^62^. The fine-tuning was done on a GeForce RTX 2080 Ti graphics card, and comprised 200 iterations. Each iteration involved gradient computations in the forward pass, and network weight updates in the backward pass. The weights of the Inception V3’s convolutional base were frozen to prevent them from being updated during the fine tuning; the weights that were updated were those of a 1024-dimensional fully connected layer and the final 4-dimensional classification head that were attached to the convolutional base. The cross-entropy loss and accuracy curves were used to track the training progress, which took approximately 4 hours. Upon convergence, the fine-tuned classifier performance was measured using both F1 accuracy metric and a confusion matrix; as an example, the element of the matrix at position *(A, B)* would represent the count of test set images that belong to class *A,* but which were wrongly predicted as belonging to class *B.* In addition, the distribution over classifier confidence scores for each class was used to visually assess the classifier performance. The figures for the confusion matrix and distribution over scores are shown in Fig. 5A,C.

#### Unsupervised seafloor classification

##### Training data generation strategies

Unsupervised classification groups similar images in feature space into clusters. This has the benefit of requiring no hand labeling. However, a subset of images is required as training examples to fit the unsupervised classifier. This is because executing the classification has a direct implication for the needed computer memory and execution time, and scales at least quadratic to the number of samples^63^. Therefore, four sampling strategies were evaluated with the aim of identifying the optimal method for sampling a representative training set comprising *n* = *5000* images. The sample size *n* was chosen to correspond to the same number of images, as was done in the semi-automated labeled image generation mentioned above (12.5% of the dataset). In addition, entropy was extracted from each image and chosen as a feature in two sampling strategies. This is because the variation in the content of the images used here is mostly in terms of distribution of Mn-nodules, which was assumed to be well captured by entropy. Also, entropy is fairly computationally cheap to extract, which was convenient for an evaluation experiment. Other seafloor images might require and work better with another image feature.

First, random sampling was used to generate the training set *T*^*(random)*^ by assigning each image an equal likelihood of being drawn. Second, spatially uniform sampling was used to uniformly sample the geographic extent of the working area. The training set *T*^*(spatial)*^ was generated by binning the geographic coordinates representing each image into *n* bins, and drawing an image randomly from each bin. Third, stratified cluster-based sampling used k-means clustering to stratify the images based on their entropy features. The training set *T*^*(stratified)*^ comprising *n* images was generated by uniformly sampling each stratum. Finally, probabilistic weighted re-sampling was used to reduce imbalance by oversampling the under-represented regions of a probability density function (pdf). The pdf was generated by fitting a one-dimensional gaussian mixtures model using the entropy features extracted from all images as datapoints. The assumption is that these datapoints were drawn from a probability distribution comprising a mixture of a finite number of gaussians (20 in this case). The pdf was then generated by inferring the parameters of this probability distribution during model fitting. The training set *T*^*(probabilistic)*^ was then generated by weighted random sampling, where each image was assigned a weight equal to its density under the inverted version of the pdf.

##### Training k-means classifier for each training data generation strategy

The feature vectors for each training set in $\left\{{T}^{(random)}, {T}^{(spatial)} , {T}^{(stratified)}, {T}^{(probabilistic)}\right\}$ were retrieved from the data matrix *X,* and used to separately fit a k-means classifier. The optimum number of clusters was determined by computing the silhouette score^64^ for distinct number of clusters ranging between 2 and 20. The number of clusters that resulted in the highest score was chosen as the optimum.

##### Determination of the optimal training data generation strategy

Each training data generation strategy was evaluated based on two metrics. These include time-to-fit, and the quality of the resulting clusters. The silhouette score metric was used to quantify the quality of clusters by measuring how dense the elements in each cluster are relative to other clusters. Time-to-fit was measured in number of seconds it took the used computer to sample the training data and fit the classifier until convergence. The sampling strategy corresponding to the classifier that resulted in the highest silhouette score and a comparable short computation time was chosen as the optimal strategy.

### Mapping the spatial distribution of seafloor classes

The georeferenced coordinates of each image were used to visualize each photo location along the OFOS tow-tracks, color coded by the corresponding seafloor class A to D. The seafloor classification maps for the German and Belgian license area were generated separately. In addition, maps were also generated for the German contract area, showing the seafloor classes before and after the dredge experiment.

## Supplementary Information

Supplementary Information 1.

Supplementary Information 2.

## Data Availability

The datasets presented in this study can be found online in PANGAEA through the following link https://doi.pangaea.de/10.1594/PANGAEA.935856. Intermediate data generated during the analysis is also provided in the supplementary materials as an excel file. To request the data used in this study, please contact Prof. Dr. Jens Greinert using the email address jgreinert@geomar.de.
