# Supplementary material for: Implementation of an automated workflow for image-based seafloor classification with examples from manganese-nodule covered seabed areas in the Central Pacific Ocean
Source: Sci Rep. 2022 Sep 12;12:15338. doi: 10.1038/s41598-022-19070-2 (PMC9468037; doi:10.1038/s41598-022-19070-2)
Supplement: Supplementary file 1 — Supplementary Information 1. [file 41598_2022_19070_MOESM1_ESM.pdf]

# Implementation of an automated workflow for image-based seafloor classification with examples from Manganese-nodule covered seabed areas in the Central Pacific Ocean

**Benson Mbani[1*], Timm Schoening[1], Iason-Zois Gazis[1], Reinhard Koch[2], Jens Greinert[1,3]**

[1]GEOMAR Helmholtz Center for Ocean Research – Kiel, DeepSea Monitoring Group, Wischhofstraße 1-3, 24148 Kiel, Germany

[2]Kiel University, Department of Computer Science, Ludewig-Meyn-Str. 10-12, 24098 Kiel, Germany.

[3]Kiel University, Institute of Geosciences, Ludewig-Meyn-Str. 10-12, 24098 Kiel, Germany.

*** Correspondence:**
Corresponding Author
bmbani@geomar.de

## Supplementary Information

### Detailed description of illumination and color normalization

This section provides detailed mathematical formalisms for the illumination and color normalization section of the paper, which were used to improve the visual quality of the underwater image datasets described in the paper.

First, the illumination drop-off towards the corners of each image is removed independently. The image dataset is sorted by acquisition time and split into batches comprising 50 images. For each batch, a mean image $I^{(\mu)}$ and a standard deviation image $I^{(\sigma)}$ are computed. These are used to generate the corrected image $I^{(IL)}$ using the z-score normalization:

$$I^{(IL)} = \frac{I - I^{(\mu)}}{I^{(\sigma)}}$$

Second, adaptive histogram equalization is applied to maximize contrast in each image. Therefore, the normalized histogram $H$ of $I^{(IL)}$ is computed ($|H| = 256$, $\sum_{i=0}^{255} H_i = 1$). Each pixel of color $p_c$ in $I^{(IL)}$ is then mapped to a color $q_c$ to create an image $I^{(HE)}$, where $q_c$ is determined by:

$$q_c = 255 * \sum_{i=0}^{p_c} H_i$$

Finally, color normalization is applied to each $I^{(HE)}$ to equalize all $N$=*40,678* images together to a reference illumination. The one image $\bar{I}^{(HE)}$ with the largest scale and maximum resolution (in px/cm) is chosen as a reference image (see supplementary Fig. S1):

$$\bar{I}^{(HE)} = I^{(HE)}_{\mathrm{argmax}_i\{s_i | i=0,..N-1\}}$$

The reference cumulative distribution function $G$ is computed from the histogram $\overline{H}$ of the reference image $\bar{I}^{(HE)}$:

$$G(k) = \sum_{i=0}^{k} \overline{H}_i \ , \qquad k = 0, 1, 2, 3, \dots, 255$$

Also, the source cumulative distribution function $S$ is computed from the histogram $H$ of each contrast-enhanced image $I^{(HE)}$:

$$S(k) = \sum_{i=0}^{k} H_i \ , \qquad k = 0, 1, 2, 3, \dots, 255$$

The color normalized image $I^{(IN)}$ is then formed from $I^{(HE)}$. This is done by mapping each value $r$ from the source distribution $S$, to the corresponding value $z$ that has the same probability in the reference distribution $G$:

$$z = G^{-1}\left(S(r)\right)$$

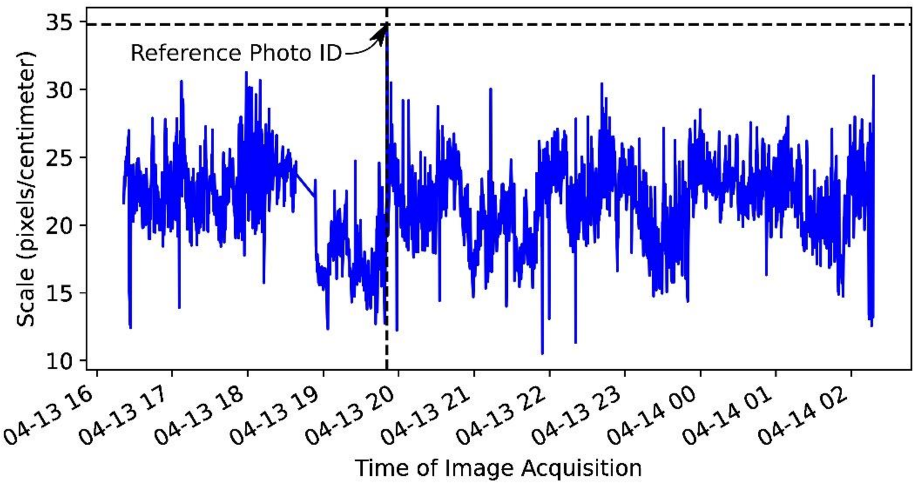


**Supplementary Figure S1: Distribution of image scales as a function of acquisition time during the OFOS dive at station 126. The reference image to be used for color normalization is chosen to have the maximum resolution.**

## Relationship between image-based seafloor classification and acoustic-derived properties

This section provides a very brief summary of the analysis of hydroacoustic data recorded during the multibeam mapping performed during cruise SO268 [1]. We follow this with a brief discussion, in which we compare instances of our Seafloor B, C and D against seafloor morphology and multibeam backscatter values obtained from the hydroacoustic analysis. Seafloor A was omitted from this comparison since it was artificial seafloor, which was formed as a result of the settling sediment plume following the dredging experiment done in the German contract area. The aim of this comparison was to further investigate the spatial distribution of our seafloor classes, and also to check if these classes are meaningful e.g., for potentially large-scale habitat classification purposes.

We clarify that this section does not provide an in-depth description of the acoustic data processing methods e.g., for interpreting geological and geomorphological characteristics for purposes of quantifying and assessing the concentration of Mn-nodules on large areas of the seabed.  For a comprehensive description and in-depth discussion of these aspects, we point interested readers to recent studies such as [2], [3], [4], [5], [6] and [7]. In addition, we note that compared to the optical images recorded by the OFOS, the resolution of our 12kHz ship-based hydroacoustic dataset that was recorded from an average of 4,280 m water depth may not be sufficient for showing fine scale variation in seafloor classes in certain applications.

Both the raw and processed bathymetry and multibeam backscatter datasets were acquired during the same cruise SO268 that also acquired the underwater optical images used in this study, and these acoustic datasets published in PANGAEA [8], [9]. These datasets were analyzed to generate seafloor morphological properties, which were compared against the seafloor classification results obtained from our image-based workflow. This was aimed at checking whether our proposed image-based workflow produces semantically meaningful seafloor substrate classes that can be potentially used for habitat classification, and also to further investigate the distribution of Mn-nodule on the seafloor.

The generated seafloor morphological properties included absolute depth, backscatter, slope, bathymetric positioning index (BPI), and ruggedness - expressed as Vector Ruggedness Measure [10]. Slope, ruggedness, and BPI raster grids were calculated using the Benthic Terrain Modeler [11] in ArcMap 10.6, with a spatial resolution of 50 x 50m [9]. A 3 x 3 neighbor was used for the slope and ruggedness calculation, whereas the BPI was calculated with an inner radius of 50m and an outer radius of 300 meters scale. The BPIs were standardized to enable comparison between the different scales and areas. In both contract areas, the same multibeam (EM122) and acquisition settings were  used [1]. Post-processing of the backscatter data utilized the Fledermaus Geocoder Toolbox (FMGT) by QPS, applying the same settings for both areas. Thus, a comparison between the two contract areas is feasible. For a more in depth description of the methods, as well as comprehensive discussion of geological reasons for the Mn-nodule distributions please see [2], [3], [4].

The five investigated seafloor morphological properties for both the German and Belgian contract areas are shown in supplementary Fig. S2. The German contract area is shallower than the Belgian area; differences in backscatter intensity are also visible in the two contract areas. In the German area, a difference in backscatter intensity can be seen between the Seafloor classes

B, C and D. Compared to seafloor regions with sparsely distributed Mn-nodules in Seafloor B, the backscatter strength is higher in regions with dense and large Mn-nodules (Seafloor C and D). This is because the acoustic signal penetrates into the sediment, and is less scattered from the rough microrelief of the Mn-nodule seafloor in patchy Mn-nodule areas (Seafloor B) compared to Seafloor C and D. This is consistent with previous findings which found an association between low backscatter strength for a seabed that is dominated by sediment, whereas higher backscatter strength was associated with medium-to-large sized Mn-nodules including outcropping rocks [12], [4], [5]. Overall, the large Mn-nodule areas (Seafloor D) returned the highest backscatter, even though in the Belgian contract area the relative difference in the backscatter strength between densely distributed and large Mn-nodules was only marginal. This could be because the two contract areas contributed disproportionately to each seafloor class. The Belgian contract area contributed significantly to both Seafloor C (96%) and Seafloor D (64%), while the German area contributed significantly to Seafloor D (35%) but insignificantly to Seafloor C (3%). This could explain why the relative difference in backscatter strength between Seafloor C and D in the German area was more pronounced compared to those in the Belgian area. The disproportionality of the Mn-nodules distribution was also observed in previous studies by [13] and [14].

Comparing the two contract areas, the median backscatter strength in the Belgian area was consistently lower than in the German area, although large and dense nodules should give a higher backscatter response than the smaller and patchy distribute occurrences. We note that for 12kHz MBES systems, the recorded backscatter signal reflects both the top of the seafloor (the top 20cm; the seafloor backscatter) but also reaches several meters into the sediment column (volume backscatter; Mitchell, 1993) [16], [17]. Especially in the German contract area, a high percentage of larger Mn-nodules (30%) is buried in the first 20 cm of the sediment column. This percentage can vary significantly (20-70%) thereby contributing to the observed backscatter intensity [18]. Opposite, the Belgian contract area has a smaller percentage of buried Mn-nodules [19]. In addition, the Belgian area lies 400m deeper than the German area, a fact that could have influenced the backscatter correction of the received signal intensity from different water depths during the acquisition or/and backscatter processing in FMGT [20] [21]. Moreover, the relative inconsistency in backscatter intensity between the two areas could be caused by the difference in spatial footprint of the images used in the classification (1.6 $m^2$), and the spatial footprint of the backscatter dataset (the beam footprint increases with depth). In a previous study, [12] point out that some level of inconsistency between in-situ observations by the OFOS and backscatter should be expected if the spatial footprints are so much different between visual and hydroacoustic observations.

Analysis of the fine BPI revealed that the large Mn- nodules were located in relatively flat regions characterized with a median BPI value of zero, while the densely distributed Mn-nodules occupied local elevations (e.g., ridges) with positive median BPI value (+1). The few sparsely distributed Mn-nodules were located in valleys with a negative median BPI value (-2). In the Belgian area, positive BPI values clearly distinguishes Seafloor C from the other classes B and D, which shows that Seafloor C occurs mainly in the local elevations of the seafloor. Correlations between the BPI and Mn-nodule distribution have also been detected in previous studies by [7], [4] and [3]. However, we emphasize that these studies were based on AUV-derived bathymetry data, which has at higher resolution than our ship-based bathymetric data.

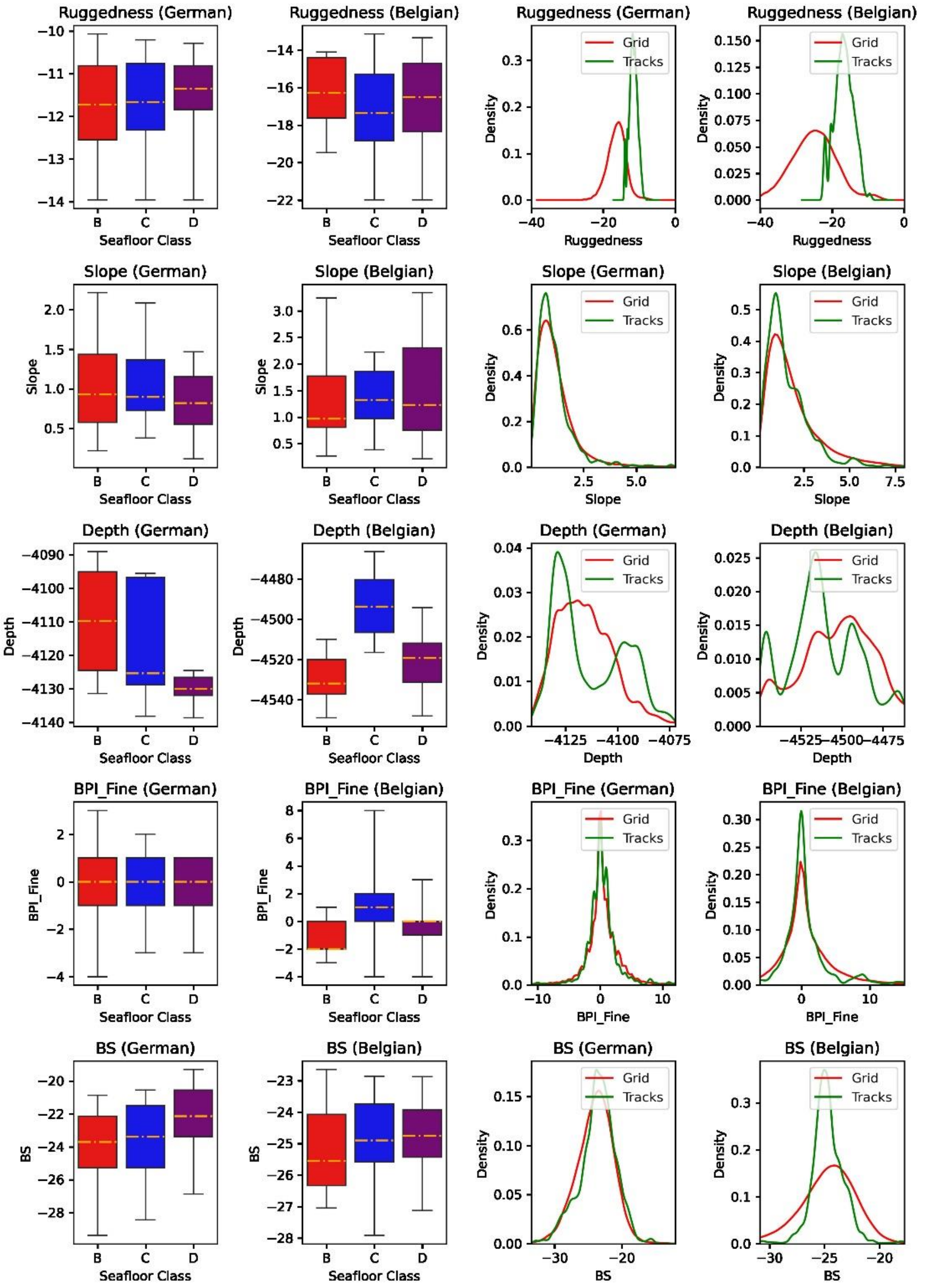


**Supplementary Figure S2: Box plots of bathymetric derivatives and MBES backscatter (BS) grouped by the respective seafloor classes. Also shown are distributions of the derivatives as sampled along the deployment tracks, compared to those within a raster grid covering the spatial extent of each visually inspected area. Overall, the distributions show that the sampling was representative. The classification confidence score threshold was set to 0.6 which represented 80% of the images. At this threshold, the proportion of images in the seafloor classes B, C and D was 13913, 132, 4059 in the German area and 260, 3599, 8015 in the Belgian area, respectively. Class A was omitted since it predominantly occurred in the German area after the dredge experiment (artificial seafloor).**

## SUPPLEMENTARY TABLES

### Table S1 Python libraries used in implementing AI-SCW components

| Python library | Usage |
|---|---|
| **pandas** | Processing navigation text files and managing csv files |
| **geopandas** | Geotagging images by matching acquisition time vs USBL navigation data |
| **contextily** | Providing basemaps for cartographic visualization on jupyter notebooks |
| **tensorflow-gpu** | Fine-tuning and inferencing Inception V3 convolutional neural network |
| **cartopy** | Cartographic visualization on jupyter notebooks |
| **scikit-learn** | Machine learning and dimensionality reduction |
| **ipython** | Rendering rich text on jupyter notebooks |
| **shapely** | Computational geometry during laser point detection |
| **matplotlib** | Visualization and plotting |
| **imageio** | Reading image datasets into memory |
| **seaborn** | Visualization and plotting on top of matplotlib |
| **tensorflow-hub** | Loading pre-trained Inception V3 model weights |
| **pillow** | Image processing |
| **rasterio** | Reading geospatial raster files such as bathymetry, backscatter e.t.c |
| **scikit-image** | Image processing |

**Table S2 Specific python scripts for running each component of AI-SCW. Both the scripts and detailed usage guides are located in the project's GitLab repository (https://git.geomar.de/open-source/AI-SCW)**

| AI-SCW Task | Python script |
|---|---|
| **Georeferencing images** | python create_table_of_geotagged_coordinates.py |
| **Laser point detection** | python detect_lasers.py<br><br>python interpolate_non_detected_lasers.py |
| **Illumination normalization** | python pixelwise_normalize.py<br><br>python adaptive_histogram_normalize.py |
| **Standardizing spatial footprint** | python rescale_and_crop_all_images.py |
| **Color balancing by reference image-based histogram matching** | python color_balance_center_cropped_images.py |
| **Extracting texture features** | python extract_texture_features.py |
| **Semi-automated image annotation** | python semi_automatically_generate_labels.py |
| **Fine-tuning inception V3 convolutional neural network** | python fine_tune_CNN_to_create_seafloor_classifier.py |
| **Classify each image using the fine-tuned Inception V3** | python classify_each_photo_using_fine_tuned_classifier.py |
| **Generating performance metrics of the fine-tuned inception v3** | python evaluate_performance_of_fine_tuned_cnn.py |
| **Evaluating sampling strategies for unsupervised classification** | python evaluate_unsupervised_seafloor_classifiers.py |

## REFERENCES


1. Haeckel, M. & Linke, P. RV SONNE Fahrtbericht/Cruise Report SO268 - Assessing the Impacts of Nodule Mining on the Deep-sea Environment: NoduleMonitoring, Manzanillo (Mexico) – Vancouver (Canada), 17.02. – 27.05.2019. GEOMAR Report, N. Ser. 059 . GEOMAR Helmholtz-Zentrum für Ozeanforschung Kiel, Kiel, Germany, 359 + Appendix (in all 802) pp. https://oceanrep.geomar.de/54402/ (2021) doi:10.3289/GEOMAR_REP_NS_59_20.

2. Joo, J. et al. Seabed Mapping Using Shipboard Multibeam Acoustic Data for Assessing the Spatial Distribution of Ferromanganese Crusts on Seamounts in the Western Pacific. Minerals **10**, 155 (2020).

3. Gazis, I.-Z., Schoening, T., Alevizos, E. & Greinert, J. Quantitative mapping and predictive modeling of Mn nodules' distribution from hydroacoustic and optical AUV data linked by random forests machine learning. Biogeosciences **15**, 7347–7377 (2018).

4. Gazis, I.-Z. & Greinert, J. Importance of Spatial Autocorrelation in Machine Learning Modeling of Polymetallic Nodules, Model Uncertainty and Transferability at Local Scale. Minerals **11**, 1172 (2021).

5. Kuhn, T. & Rühlemann, C. Exploration of Polymetallic Nodules and Resource Assessment: A Case Study from the German Contract Area in the Clarion-Clipperton Zone of the Tropical Northeast Pacific. Minerals **11**, 618 (2021).

6. Wang, M. et al. Using multibeam backscatter strength to analyze the distribution of manganese nodules: A case study of seamounts in the Western Pacific Ocean. Applied Acoustics **173**, 107729 (2021).

7. Peukert, A. et al. Understanding Mn-nodule distribution and evaluation of related deep-sea mining impacts using AUV-based hydroacoustic and optical data. Biogeosciences **15**, 2525–2549 (2018).

8. Gazis, I.-Z., Urban, P. & Haeckel, M. Swath sonar multibeam EM122 bathymetry raw data during SONNE cruises SO268/1 and SO268/2, German License Area in Clarion Clipperton Zone, Pacific. GEOMAR - Helmholtz Centre for Ocean Research Kiel (2020) doi:10.1594/PANGAEA.919755.

9. Gazis, I.-Z. Processed EM122 multibeam swath bathymetry collected during SONNE cruise SO268/1 inside the German License Area in Clarion Clipperton Zone, Pacific. GEOMAR - Helmholtz Centre for Ocean Research Kiel (2020) doi:10.1594/PANGAEA.915764.

10. Sappington, J. M., Longshore, K. M. & Thompson, D. B. Quantifying Landscape Ruggedness for Animal Habitat Analysis: A Case Study Using Bighorn Sheep in the Mojave Desert. wild **71**, 1419–1426 (2007).

11. Walbridge, S., Slocum, N., Pobuda, M. & Wright, D. J. Unified Geomorphological Analysis Workflows with Benthic Terrain Modeler. Geosciences **8**, 94 (2018).

12. Machida, S. et al. Visualisation method for the broad distribution of seafloor ferromanganese deposits. Marine Georesources & Geotechnology **39**, 267–279 (2021).

13. Stackeelberg, U. & Beiersdorf, H. The formation of manganese nodules between the Clarion and Clipperton fracture zones southeast of Hawaii - ScienceDirect. https://www.sciencedirect.com/science/article/abs/pii/002532279190113I (1991).

14. Hein, J. R. & Koschinsky, A. 13.11 - Deep-Ocean Ferromanganese Crusts and Nodules. in Treatise on Geochemistry (Second Edition) (eds. Holland, H. D. & Turekian, K. K.) 273–291 (Elsevier, 2014). doi:10.1016/B978-0-08-095975-7.01111-6.

15. Mitchell, N. C. A model for attenuation of backscatter due to sediment accumulations and its application to determine sediment thicknesses with GLORIA sidescan sonar. Journal of Geophysical Research: Solid Earth **98**, 22477–22493 (1993).

16. Schneider von Deimling, J. et al. A low frequency multibeam assessment: Spatial mapping of shallow gas by enhanced penetration and angular response anomaly. Marine and Petroleum Geology **44**, 217–222 (2013).

17. Hillman, J. I. T. et al. Validation of automated supervised segmentation of multibeam backscatter data from the Chatham Rise, New Zealand. Mar Geophys Res **39**, 205–227 (2018).

18. Kuhn, T., Rühlemann, C. & Wiedicke-Hombach, M. Development of Methods and Equipment for the Exploration of Manganese Nodules in the German License Area in the Central Equatorial Pacific. 4 (2011).

19. Schoening, T. & Gazis, I.-Z. Sizes, weights and volumes of poly-metallic nodules from box cores taken during SONNE cruises SO268/1 and SO268/2. GEOMAR - Helmholtz Centre for Ocean Research Kiel (2019) doi:10.1594/PANGAEA.904962.

20. Lurton, X. et al. Backscatter measurements by seafloor-mapping sonars: guidelines and recommendations. A collective report by members of the GeoHab Backscatter Working Group 1–200 (2015).

21. Malik, M. et al. Results from the First Phase of the Seafloor Backscatter Processing Software Inter-Comparison Project. Geosciences **9**, 516 (2019).
